# Construction of High-Density Genetic Maps and Detection of QTLs Associated With Huanglongbing Tolerance in Citrus

**DOI:** 10.3389/fpls.2018.01694

**Published:** 2018-11-27

**Authors:** Ming Huang, Mikeal L. Roose, Qibin Yu, Dongliang Du, Yuan Yu, Yi Zhang, Zhanao Deng, Ed Stover, Frederick G. Gmitter

**Affiliations:** ^1^Citrus Research and Education Center, Institute of Food and Agricultural Sciences, University of Florida, Lake Alfred, FL, United States; ^2^Department of Botany and Plant Sciences, University of California, Riverside, Riverside, CA, United States; ^3^Gulf Coast Research and Education Center, University of Florida, Wimauma, FL, United States; ^4^United States Horticultural Research Laboratory, Agricultural Research Service, United States Department of Agriculture, Fort Pierce, FL, United States

**Keywords:** *Candidatus* Liberibacter, genetic map, F_1_ population, genotyping-by-sequencing, *Poncirus*, QTL mapping, SNP

## Abstract

Huanglongbing (HLB), or citrus greening, is the most devastating disease in citrus worldwide. Commercial citrus varieties including sweet orange (*Citrus sinensis*) are highly susceptible to HLB, and trifoliate orange (*Poncirus trifoliata*, a close *Citrus* relative) is widely considered resistant or highly tolerant to HLB. In this study, an intergeneric F_1_ population of sweet orange and trifoliate orange was genotyped by Genotyping-by-Sequencing, and high-density SNP-based genetic maps were constructed separately for trifoliate orange and sweet orange. The two genetic maps exhibited high synteny and high coverage of the citrus genome. Progenies of the F_1_ population and their parents were planted in a replicated field trial, exposed to intense HLB pressure for 3 years, and then evaluated for susceptibility to HLB over 2 years. The F_1_ population exhibited a wide range in severity of HLB foliar symptom and canopy damage. Genome-wide QTL analysis based on the phenotypic data of foliar symptom and canopy damage in 2 years identified three clusters of repeatable QTLs in trifoliate orange linkage groups LG-t6, LG-t8 and LG-t9. Co-localization of QTLs for two traits was observed within all three regions. Additionally, one cluster of QTLs in sweet orange (linkage group LG-s7) was also detected. The majority of the identified QTLs each explained 18–30% of the phenotypic variation, indicating their major role in determining HLB responses. These results show, for the first time, a quantitative genetic nature yet the presence of major loci for the HLB tolerance in trifoliate orange. The results suggest that sweet orange also contains useful genetic factor(s) for improving HLB tolerance in commercial citrus varieties. Findings from this study should be very valuable and timely to researchers worldwide as they are hastily searching for genetic solutions to the devastating HLB crisis through breeding, genetic engineering, or genome editing.

## Introduction

Huanglongbing (HLB), commonly known as citrus greening, is the most devastating disease in citrus plantations worldwide. Since the first identification of the disease in Florida in 2005, HLB has spread throughout the state, and is now found in most states in the United States where citrus is grown. It is rapidly spreading throughout many of the world’s production areas, as well. Due to widespread infection and lack of effective management strategy, Florida’s nearly $11 billion citrus industry has experienced a rapid and continuous decline. The production of sweet orange dropped from 240 million boxes in 2004 to 45 million in 2018 (Florida Citrus Commission^1^).

Huanglongbing-diseased trees develop leaves with characteristic blotchy mottle, the shoots are stunted and yellowing, and the canopy and branches gradually dieback as the disease progresses. These symptoms are associated with the host limitations for photoassimilate transport and nutrient uptake induced by the disease, and finally can lead to tree death (Blaustein et al., 2018). HLB is generally considered to be caused by three species of *Candidatus* Liberibacter, of which *Candidatus* Liberibacter asiaticus (*C*Las) is the most widespread and virulent species and is the only species reported in the citrus industry of United States. *C*Las is a heat-tolerant Gram-negative bacterium, resides only in the phloem of plant hosts, and is vectored by the sap-sucking Asian citrus psyllid (ACP, *Diaphorina citri*) (Wang N.A. et al., 2017). As an obligate and insect-transmitted plant pathogen, *C*Las attacks all species and hybrids in the genus of *Citrus* as well as some closely related genera (Ramadugu et al., 2016). Most commercial citrus cultivars are highly susceptible to HLB (Stover et al., 2016a,b). Within cultivated citrus, high levels of tolerance to HLB were mostly found in some types that are commonly used as rootstocks, such as trifoliate orange and some of its hybrids (Albrecht and Bowman, 2011, 2012; Ramadugu et al., 2016).

Until now, very little is known about the molecular mechanism of pathogenesis of *C*Las (Martinelli and Dandekar, 2017). As to understanding the genetic architecture of citrus resistance or susceptibility to HLB, there has been no compelling progress, and no QTL related to *C*Las infection or HLB tolerance responses has been reported. Moreover, so far there is no sustainable management and control of HLB where it is endemic. Breeding and development of HLB resistant or tolerant cultivars is widely regarded to be the most practical strategy to support long-term control of this severe disease in the field. The reports of variability for tolerance of or sensitivity to HLB within citrus and its relatives encourages breeding and selection for tolerant or possibly resistant genotypes (Richardson and Hall, 2013; Ramadugu et al., 2016; Killiny et al., 2017; Miles et al., 2017). Traditional breeding through crossing elite cultivars with resistant materials can achieve this objective. However, the introgression of resistant germplasm into elite cultivars will likely require multiple rounds of backcrossing to recover desirable commercial traits. In addition, the breeding cycle for citrus ranges from 5 to 10 years, and the rescue of the citrus industries through HLB-resistant or tolerant cultivars demands considerable urgency. Identification of QTLs associated with resistance or tolerance to HLB in citrus can facilitate more rapid development of resistant cultivars through marker-assisted selection or genome editing.

To identify QTLs associated with phenotypic traits, a genetic map with high resolution and fine accuracy is crucial. Nowadays, the wide application of high-throughput genome sequencing and efficient SNP genotyping allows the construction of genetic maps with numerous markers at an acceptable cost (Deschamps et al., 2012). While initially confined to annual herbaceous plants, high-density genetic mapping is increasingly extended to perennial woody plants. Using high-throughput genotyping, the saturation of genetic maps has been greatly improved for some citrus species, such as sweet orange, mandarin and pummelo (Lyon, 2008; Ollitrault et al., 2012a; Shimada et al., 2014; Guo et al., 2015; Yu et al., 2016; Curtolo et al., 2017; Imai et al., 2017). However, in comparison with well-studied model and agronomic plants, citrus lags in development of high-density, high-resolution genetic maps with fine accuracy and precision. Moreover, so far there is no high-density genetic map for trifoliate orange.

This study inaugurates investigation of HLB infection in citrus by repeated phenotyping of segregating populations and QTL mapping. The objectives of this study were: (1) to evaluate severity level of HLB disease among a field population exposed to intense HLB pressure using two phenotypic traits, foliar symptoms and canopy damage; (2) to construct high-density genetic maps separately for trifoliate orange and sweet orange in an F_1_ mapping population through Genotyping-by-Sequencing; (3) to identify QTLs associated with citrus HLB infection and responses separately in trifoliate orange and sweet orange genetic maps.

## Materials and Methods

### Plant Materials

Genotyping was carried out in an F_1_ population of 170 individuals derived from mixed intergeneric crosses between two sweet oranges (*Citrus sinensis* ‘Sanford’ and ‘Succari’) and two trifoliate oranges (*Poncirus trifoliata* ‘Argentina’ and ‘Flying Dragon’), including 79 progenies of ‘Sanford’ × ‘Argentina’, 40 progenies of ‘Succari’ × ‘Flying Dragon’, and 51 progenies of ‘Flying Dragon’ × ‘Succari’. Of the genotyping population, 86 individuals were randomly chosen as a phenotyping population, including 47 progenies of ‘Sanford’ × ‘Argentina,’ 14 progenies of ‘Succari’ × ‘Flying Dragon’, and 25 progenies of ‘Flying Dragon’ × ‘Succari’. In addition, two sweet orange varieties (‘Navel’ grafted on ‘Swingle’ and ‘Hamlin’ grafted on ‘C-35’), six trifoliate orange varieties (‘Flying-Dragon’, ‘Argentina’, and ‘Pomeroy’ grafted on ‘Volkamer’ lemon; and seedlings of ‘Rubidoux’, ‘Rich 16-6’, and ‘Large-flower’), and seedlings of ‘Volkamer’ lemon (*Citrus volkameriana*) were included as controls in the phenotyping. All progenies were clonally propagated by grafting on ‘Volkamer’ lemon in the greenhouse of the USDA/ARS in Fort Pierce, FL in 2010. Except for ‘Volkamer’ lemon seedings that had 16 replicate trees, each of the progenies and control varieties had eight clonal replicate trees. A completely randomized experiment of 768 trees was established in a field trial at the USDA-ARS in Fort Pierce in 2011. The planting consisted of eight rows oriented south-north, with 4 m × 1.5 m planting distances. Guard citrus trees not analyzed in this study were planted at the ends of each row. The field population trees were irrigated and fertilized with professional practices, but pesticides were not applied during the study, to encourage psyllid population increase, feeding, colonization, and inoculation of *C*Las to the trees. Maintenance of the field trees was as described for a nearby experiment on the same farm (Richardson et al., 2011).

### Detection of *C*Las Infection

As reported in other concurrent studies (Lewis-Rosenblum et al., 2015; Ramadugu et al., 2016), the HLB disease pressure was high in the field trial at this location, which caused homogeneous inoculation of *C*Las naturally, providing excellent conditions to evaluate trees under natural disease spread conditions (Westbrook et al., 2011; Richardson and Hall, 2013). During the period of HLB disease evaluation, the status of *C*Las infection for each tree was diagnosed using the TaqMan label-based multiplex real-time PCR method (Li W.B. et al., 2006, 2008). At least four fully expanded mature leaves were randomly collected from different branches and different quadrants of each tree. The midribs were separated from the leaves and cut into small pieces for DNA extraction using the CTAB method (Aldrich and Cullis, 1993). Real-time qPCR was performed on an ABI 7500 thermocycler with probes specific to *C*Las 16S ribosomal gene and citrus cytochrome oxidase gene. The mean cycle threshold (C*t*) values of qPCR for direct *C*Las detection were normalized with C*t* values of the corresponding host plant gene. Trees with C*t* value under 33 were considered to be HLB-positive (Albrecht and Bowman, 2012).

### Evaluation of HLB Disease

Evaluations of HLB disease symptoms were performed twice per year from 2015 to 2016 in September and October, which is the optimal time for HLB disease evaluation considering both the growth condition of citrus (*Poncirus* and some of its hybrids are deciduous or semi-deciduous) and the period of the most evident disease symptoms. At each time of disease evaluation, the visual evaluation of foliar symptom and canopy damage was conducted twice per tree on each side along the tree rows. All evaluations were carried out by the same individual researcher. Foliar disease symptom severity was assessed on a 6-point scale visual rating for comprehensive typical HLB symptom including mottled rugose leaves and yellow shoots: 0 = no symptom for whole tree, 1 = isolated (less than 1/10 of tree) slight symptom, 2 = partly (approximately 1/3 of tree) slight symptom or isolated severe symptom, 3 = mostly (approximately 2/3 of tree) slight symptom or partly severe symptom, 4 = mostly severe symptom, 5 = severe symptom for whole tree. The slight foliar symptom rating refers to trees with leaves that are slightly mottled, pale or leathery, while the severe symptom rating refers to severely blotchy mottled, rugose or leathery. Canopy damage severity was also assessed on a 6-point scale visual rating basing on dieback, defoliation or stunting: 0 = full tree canopy without apparent dieback or defoliation or stunting, 1 = full tree canopy with isolated short dieback or very slight defoliation, 2 = partly short dieback or isolated long dieback or slight defoliation or stunting, 3 = mostly short dieback or partly long dieback or moderate defoliation or stunting, 4 = mostly long dieback or main branch dieback or severe defoliation or stunting, 5 = trunk dieback or whole tree dead. The mean rating score for each genotype in each year was the averaged rating score of all replicate trees at the two times of evaluation.

### Phenotypic Analysis

Descriptive statistics of all phenotypic data were calculated using IBM SPSS (Statistical Package for Social Sciences) Statistics 17.0. Significant differences between phenotypic data were declared with *p* ≤ 0.05 by Student’s *t*-test. To evaluate whether the data followed a normal distribution, a normality analysis by Kolmogorov–Smirnov and Shapiro–Wilk tests was performed separately for the dataset of each trait in each year. The Box-Cox transformation was performed before QTL analysis if data presented a non-normal distribution. Histograms for each trait were constructed using the mean rating score of the complete dataset. Pearson’s correlation coefficients were calculated between year-means for each phenotypic trait.

### Genotyping by Sequencing

Genomic DNA of each citrange hybrid and parent was extracted using a modified CTAB method (Aldrich and Cullis, 1993), Genomic DNA was digested with PstI restriction endonuclease and then processed into restriction site associated DNA (RAD) libraries according to a previously described protocol (Baird et al., 2008). The constructed RAD libraries were sequenced on an Illumina HiSeq2000 platform following the manufacturer’s protocol. Library construction, sequencing and SNP calling were provided by Floragenex (Floragenex Inc., Portland, OR, United States). SNP calling of parents and progenies was performed using the Clementine mandarin genome v1.0^2^ as a reference. Genotypes at each locus were determined using the VCF Popgen Pipeline version 4.0 to generate a customized VCF 4.1 (variant call format) database with parameters set as follows: minimum allele frequency for genotyping 0.075, minimum Phred score 15, minimum depth of sequencing coverage of 12, and minimum 75% of individuals with the specific genotype call. The marker configuration codes “lm × ll” and “nn × np” were used to represent markers that were heterozygous only in one of the parents, and code “hk × hk” to represent markers that heterozygous in both parents.

### Linkage Analysis and Construction of Parental Linkage Maps

Due to asexual means of evolution (somatic mutations), molecular polymorphism among sweet orange cultivars is very rare (Ollitrault et al., 2012b); likewise very low polymorphism was found between the trifoliate orange varieties, so all F_1_ progenies from different crosses between the two genera were considered as a single family, after excluding the inconsistent marker loci between parental varieties. To ensure high quality of linkage mapping for each parent, SNP markers segregating from only one of the parents (with configuration codes “lm × ll” and “nn × np”) were selected to construct the linkage maps; markers with code “hk × hk” were not considered in this study. SNP markers matching the following criteria were excluded from linkage analysis: (1) had a missing genotype in more than 10% of progenies; (2) had a missing genotype for one of the parents; (3) had inconsistent genotypes among different parental sweet oranges or trifoliate oranges; (4) had homozygous genotypes for both parents; (5) had heterozygous genotype for both parents; (6) had genotypes in F_1_ progeny not expected for the parental genotypes; (7) had no segregation in F_1_ progeny. Segregation distortion was tested by χ^2^ conformity tests against the Mendelian segregation ratio of 1:1. Because the method of linkage analysis was based upon a test for independence of logarithm of the odds (LOD) scores that is not affected by segregation distortion, markers with certain skewed segregation were included in linkage analysis referring to previous studies on citrus (Bernet et al., 2010; Ollitrault et al., 2012a; Raga et al., 2012). Linkage analyses were performed using JoinMap 4.1 (Van Ooijen, 2011). Linkage mapping was performed under a two-way pseudo-testcross scheme (Grattapaglia and Sederoff, 1994) with two separate datasets, one with markers segregating from the trifoliate orange and the other one with markers segregating from sweet orange. Markers with identical segregation patterns or segregating similarity higher than 98% were excluded from the linkage groups. Phases of the linked marker loci were automatically detected by the software. Linked markers were grouped using the independence LOD with a threshold LOD score of 4.0 and a maximum recombination fraction (θ) of 0.4. Map distances were estimated in centiMorgans (cM) using the regression mapping algorithm and the Kosambi mapping function. The linkage groups were numbered according to the corresponding scaffold number of the reference Clementine mandarin genome, and marker names were composed of the scaffold number and SNP position on the reference genome. The genetic map was drawn using the MapChart 2.2 program (Voorrips, 2002).

### QTL Mapping

QTL mapping was carried out on two parental maps separately using MapQTL 6 under the backcross model by composite interval mapping (CIM) (van Ooijen, 1992). Phenotypic data were analyzed separately for each trait in each year. The LOD thresholds to declare a significant QTL for each trait were determined via permutation tests using 1,000 permutations at a genome-wide significance level of 0.90. The CIM analysis was performed in 1 cM steps to detect significant QTLs with a LOD score higher than the threshold. The nearest marker to the likelihood peak of each significant QTL was selected as a cofactor to perform restricted multiple QTL mapping (rMQM). If the LOD value linked with a cofactor fell below the threshold during rMQM mapping, the cofactor was removed, and the analysis repeated. This process was continued until the cofactor list remained stable. Kruskal–Wallis (KW) test was also used to provide complementary validation for significant genotypic means. Each significant QTL was characterized by its LOD score, percentage of explained phenotypic variation, confidence interval (in cM) corresponding to threshold LOD score and extension region at either side of the likelihood peak until the LOD score dropped to 2.0. QTLs that showed clearly overlapping confidence intervals were considered as co-localized. The position of QTLs on the genetic map was drawn using the MapChart 2.2 program (Voorrips, 2002).

## Results

### RAD Sequencing and SNP-Based Genotyping

Four parental varieties (‘Argentina’ and ‘Flying Dragon’ trifoliate orange; ‘Sanford’ and ‘Succari’ sweet orange) and 170 F_1_ progenies were processed for RAD sequencing on the NGS Illumina platform. Except one progeny, all had acceptable quality of sequencing reads. The average number of reads was 8.14E6 and 7.37E6 for trifoliate orange and sweet orange, respectively. The read counts for the 169 F_1_ progenies ranged from 1.05E6 to 14.91E6, with an average of 4.53E6 per progeny. After alignment with the reference genome, the average number of read clusters was 1.83E5 and 1.47E5 for trifoliate oranges and sweet oranges, respectively, and the numbers of read clusters for the F_1_ progenies ranged from 0.32E5 to 2.79E5 and averaged to be 1.03E5 (the detailed data are available in Supplementary File S1).

In total, 51,687 putative SNP loci were determined, of which 55.6% were transitions and 44.4% were transversions. Excluding SNP loci without calls, 96.7% of the genotyped SNP loci were identical between ‘Argentina’ and ‘Flying Dragon’ trifoliate orange, and 98.2% were identical between ‘Sanford’ and ‘Succari’ sweet orange (the detailed data are available in Supplementary File S2). Considering the low levels of genetic diversity found between the trifoliate orange parents and between the sweet orange parents, all the F_1_ progenies from different crosses were combined as a single family. After stringent filtering, a total of 3,861 high quality SNP markers with configuration code “lm × ll” or “nn × np” were retained for genetic map construction, of which 1,408 SNP markers were heterozygous in trifoliate orange and 2,453 SNP markers were heterozygous in sweet orange.

### Genetic Linkage Map Construction and Evaluation

**FIGURE 1:**
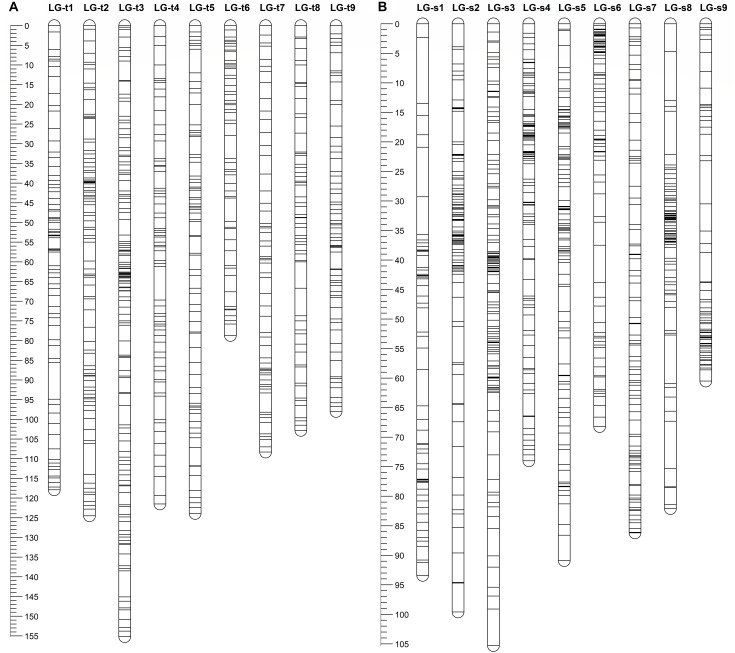
Distribution of markers with unique loci in the genetic linkage map of trifoliate orange **(A)** and sweet orange **(B)**. The nine linkage groups correspond to the nine major scaffolds of the Clementine mandarin genome. Map distances in centiMorgans (cM) are indicated by the ruler at the left.

After eliminating markers with identical or similar segregation patterns that accounted for 62% of the filtered markers, all the remaining SNP markers in each dataset were grouped under the threshold LOD score of 4.0 into nine linkage groups, which is consistent with the haploid number of chromosomes in citrus. Additionally, all nine linkage groups conserved their integrity up to LOD of 10 for both parents. Finally, for trifoliate orange, a total of 647 high-quality SNP markers were mapped on nine linkage groups with unique loci, spanning a total genetic length of 1030.8 cM, with an average inter-locus distance of 1.59 cM (Table 1 and Figure 1A). The number of markers within each linkage group ranged from 52 (for LG-t6) to 125 (for LG-t3), spanning a genetic distance ranging from 78.7 (for LG-t6) to 155.2 cM (for LG-t3). In total, 85.5% of the inter-locus gaps on the genome-wide genetic map were smaller than 3 cM and no gap was larger than 10 cM. For sweet orange, 754 high-quality SNP markers with unique loci were mapped into nine linkage groups, spanning a total genetic length of 760.2 cM with an average inter-locus distance of 1.01 cM (Table 1 and Figure 1B). The number of markers within each linkage group ranged from 57 (for LG-s1) to 118 (for LG-s3), spanning a genetic distance ranging from 60.5 (for LG-s9) to 105.3 cM (for LG-s3). Of the inter-locus gaps on the whole genetic map, 93.3% were smaller than 3 cM and only one gap was larger than 10 cM (11.2 cM on LG-s1).

**Table 1:** Summary of the genetic linkage maps of trifoliate orange and sweet orange.

Linkage group		Reference genome	Number of marker loci		LG length (cM)		Average inter-locus distance (cM)		Number of syntenic markers	
Trifoliate orange	Sweet orange	Clementine mandarin	Trifoliate orange	Sweet orange	Trifoliate orange	Sweet orange	Trifoliate orange	Sweet orange	Trifoliate orange	Sweet orange
LG-t1	LG-s1	Scaffold_1	70	57	117.9	93.4	1.68	1.64	70	57
LG-t2	LG-s2	Scaffold_2	87	89	124.5	99.6	1.43	1.12	87	89
LG-t3	LG-s3	Scaffold_3	125	118	155.2	105.3	1.24	0.89	125	118
LG-t4	LG-s4	Scaffold_4	64	94	121.5	74.0	1.90	0.79	64	88
LG-t5	LG-s5	Scaffold_5	72	98	123.9	90.9	1.72	0.93	72	98
LG-t6	LG-s6	Scaffold_6	52	77	78.7	68.2	1.51	0.89	52	77
LG-t7	LG-s7	Scaffold_7	57	85	108.3	86.2	1.90	1.01	47	73
LG-t8	LG-s8	Scaffold_8	58	76	102.8	82.1	1.77	1.08	58	64
LG-t9	LG-s9	Scaffold_9	62	60	98.0	60.5	1.58	1.01	62	60
		Total	647	754	1030.8	760.2	1.59	1.01	637	724

*The correspondence between linkage groups and syntenic scaffolds of Clementine mandarin genome was based on the location of most mapped markers.*

**FIGURE 2:**
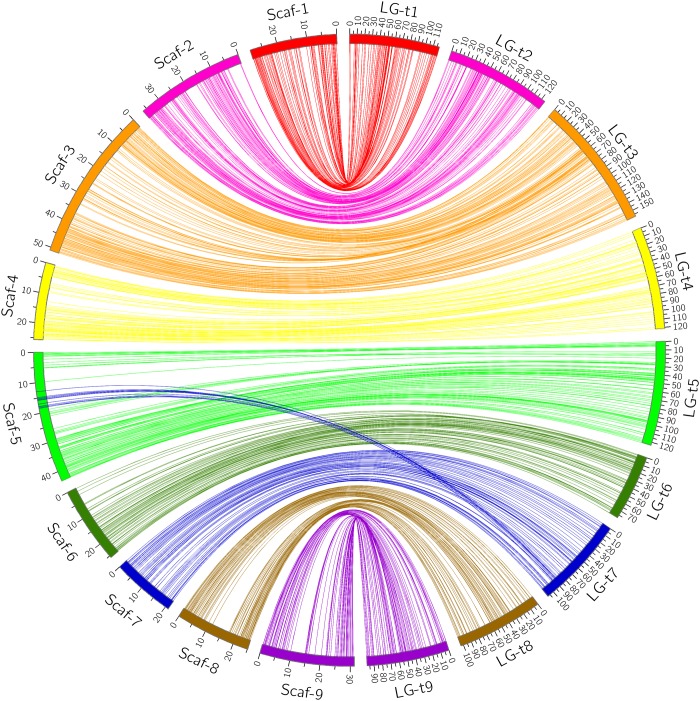
Conservation of synteny and linear order of markers between trifoliate orange genetic map and Clementine mandarin genome via circle diagram.

**FIGURE 3:**
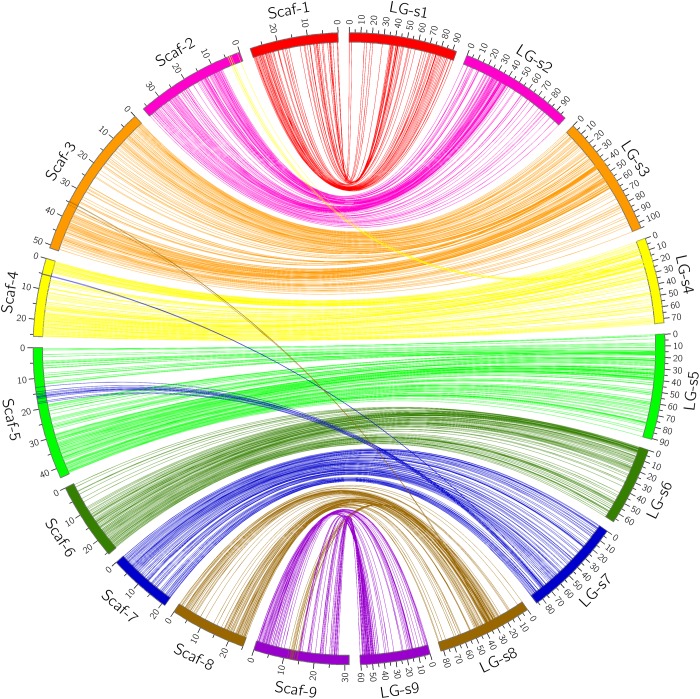
Conservation of synteny and linear order of markers between sweet orange genetic map and Clementine mandarin genome via circle diagram.

Through alignment, all SNP markers on the two genetic maps were successfully mapped onto nine major scaffolds of the Clementine mandarin genome. For trifoliate orange, except for 10 markers of LG-t7 which mapped on Scaffold_5, all other 637 markers were mapped onto syntenic scaffolds (Table 1 and Figure 2). The overall coverage ratio of mapped markers on the Clementine genome was 98.6%, and the coverage ratio on each scaffold ranged from 95.5 (for LG-t7) to 99.7% (for LG-t5). For sweet orange, except for a total of 30 markers on LG-s4, LG-s7 and LG-s8, all other 724 markers were mapped onto syntenic scaffolds (Table 1 and Figure 3). The overall coverage ratio of mapped markers on the Clementine genome was 95.0%, and the coverage ratio on each scaffold ranged from 87.7 (for LG-s9) to 99.3% (for LG-s8). Only some minor discrepancies were observed between the genetic maps and the Clementine genome, possibly due to genetic divergence among different citrus species, potential errors in the current linkage grouping, or erroneous assemblies in the reference genome. Collinear analysis of the consensus between the genetic map and the Clementine genome via a dot-plot diagram not only showed variations of the ratios between genetic distance to physical distance, but also clearly revealed high consensus between the genetic maps of trifoliate orange and sweet orange (Supplementary Figure S1).

### Phenotyping of HLB Infection

The phenotyping population consisted of 86 F_1_ progenies randomly chosen from the above-mentioned mapping population, as well as six trifoliate oranges, two sweet oranges, and the rootstock seedings, with eight clonal replicate trees for each of individuals. After exposure to intense HLB pressure for 3 years in a replicated field trial, all phenotyped trees were diagnosed for *C*Las infection by the TaqMan label-based multiplex real-time PCR during the period of disease evaluation. As shown in Table 2, relatively few trees of trifoliate orange varieties were determined to be HLB-positive; in contrast, most trees of sweet orange varieties were HLB-positive. Meanwhile, F_1_ progenies were mostly infected by *C*Las in both 2015 and 2016. For the HLB-negative genotypes, it should be noted that these trees were not confirmed to be immune to *C*Las, but they mostly had low titers of *C*Las (C*t* value of *C*Las diagnosis between 33 and 39), which might indicate partial resistance to *C*Las infection. The results indicate that all trees of the phenotyping population were adequately inoculated with *C*Las under continuous reinfection (the detailed data are available in Supplementary File S3).

**Table 2:** Diagnosis of *C*Las infection and evaluation of severity level of HLB disease among control varieties and F_1_ progenies.

		Hamlin	Navel	Argentina	Flying-dragon	Large-flower	Pomeroy	Rich 16-6	Rubidoux	Volkamer	Progenies
		
		Sweet orange	Sweet orange	Trifoliate orange	Trifoliate orange	Trifoliate orange	Trifoliate orange	Trifoliate orange	Trifoliate orange	Rootstock	Hybrids
*C*Las	2015	83.3%	89.3%	25.0%	8.3%	15.6%	22.9%	8.3%	25.0%	78.1%	79.8%
infection^a^	2016	94.4%	100.0%	15.0%	7.6%	27.9%	33.5%	12.9%	25.0%	80.0%	87.4%
Foliar	2015	4.1 ± 0.1	3.9 ± 0.1	1.1 ± 0.1	1.7 ± 0.3	1.6 ± 0.2	1.2 ± 0.1	1.4 ± 0.1	1.3 ± 0.1	2.9 ± 0.1	3.3 ± 0.1
symptom^b^	2016	3.8 ± 0.3	3.6 ± 0.2	1.4 ± 0.2	1.9 ± 0.1	1.4 ± 0.1	1.4 ± 0.1	1.2 ± 0.1	1.4 ± 0.1	2.3 ± 0.1	3.0 ± 0.1
Canopy	2015	3.8 ± 0.3	4.5 ± 0.3	1.3 ± 0.2	2.0 ± 0.3	1.3 ± 0.1	1.3 ± 0.2	1.4 ± 0.1	1.5 ± 0.2	1.2 ± 0.1	2.7 ± 0.1
damage^b^	2016	3.7 ± 0.3	4.4 ± 0.3	1.1 ± 0.1	2.0 ± 0.3	1.3 ± 0.1	1.0 ± 0.1	1.1 ± 0.1	1.1 ± 0.1	1.1 ± 0.1	2.6 ± 0.1

*^a^The status of CLas infection for all control varieties, indicated with the percentage of HLB-positive trees diagnosed by the TaqMan label-based real-time qPCR, were based on results of replicate trees; but for F_1_ progenies, were the averaged results of 86 individuals. ^b^The rating scores and variations for all control varieties, shown as Mean ± SE (n = 8), were based on scores of replicate trees; but for F_1_ progenies, were the averaged means and variations of 86 individuals.*

**FIGURE 4:**
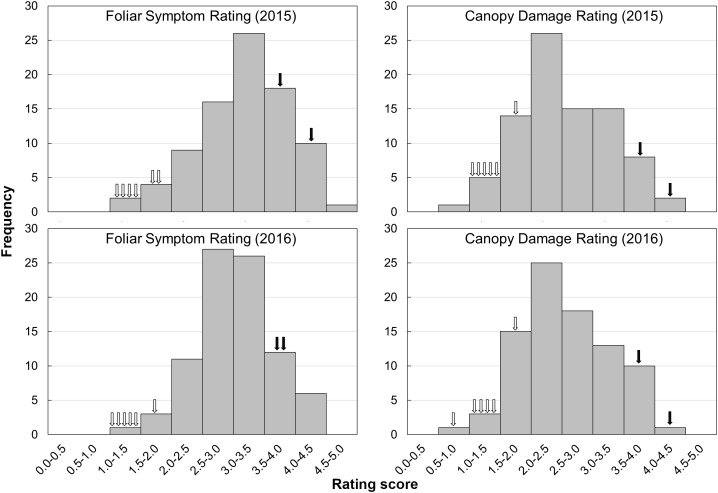
Frequency distributions of foliar symptoms rating and canopy damage rating in F_1_ progenies and parental varieties in 2 years. The hollow arrows indicate each of the trifoliate orange varieties, while the solid arrows indicate each of the sweet orange varieties.

The evaluation of HLB disease among the phenotyping population was conducted in 2015 and 2016 through repeated rating of the foliar symptom and canopy damage separately (Table 2). The ratings of foliar symptom in trifoliate oranges (ranged from 1.1 to 1.9) were significantly lower than those in sweet oranges (ranged from 3.6 to 4.1). For F_1_ progenies, the ratings of foliar symptom ranged from 1.3 to 4.7, and the average rating was 3.3 in 2015 and 3.0 in 2016. The ratings of canopy damage in trifoliate oranges (ranged from 1.0 to 2.0) were also significantly lower than those in sweet oranges (ranged from 3.7 to 4.5). For F_1_ progenies, the ratings of canopy damage ranged from 0.9 to 4.1, and the average rating was 2.7 in 2015 and 2.6 in 2016. The frequency distributions of foliar symptom and canopy damage ratings among the F_1_ progenies are illustrated in Figure 4. As shown, obvious quantitative variation of the two traits were observed in both years. The correlation coefficient of phenotypic data between 2 years was 0.77 for foliar symptom rating and 0.91 for canopy damage rating. The correlation coefficients between two traits ranged from 0.47 to 0.74 and 0.58 in average, reflecting low consistency between the traits of disease in the progenies (the detailed data are available in Supplementary File S4).

### Detection of QTLs Associated With HLB

QTLs associated with the two phenotypic traits were detected separately in two parental genetic maps for each year (Table 3). In trifoliate orange, six and three QTLs associated with foliar symptom, and three and two QTLs associated with canopy damage, were identified in 2015 and 2016, respectively. The maximum LOD score for each of the QTLs ranged from 2.7 to 5.5, explaining an estimated phenotypic variation (*R*^2^) ranging from 13.9 to 29.9%. In sweet orange, two QTLs associated with foliar symptom were identified in both 2015 and 2016, but no significant QTL associated with canopy damage was identified in either of the 2 years. The maximum LOD score for each of the QTLs ranged from 3.1 to 5.5, explaining an estimated phenotypic variation (*R*^2^) ranging from 17.3 to 29.1%. None of the QTLs alone could explain a majority of the phenotypic variation, but they collectively explained a major part. The graphics of QTL mapping are available in Supplementary Figure S2.

**Table 3:** QTLs detected separately in trifoliate orange and sweet orange genetic maps.

Trait	QTL name	Time	LG^a^	Thold LOD^b^	Max LOD^c^	QTL position^d^	*R*^2^ (%)^e^	Nearest marker^f^	Marker position^g^	Allele type^h^	KW significance^i^
Foliar symptom	FS-2015-t6a	2015	t6	2.7	4.0	43.8	20.9	6_19748604	43.8	A/G	^∗∗∗∗∗∗^
	FS-2015-t6b	2015	t6	2.7	3.9	55.4	20.8	6_21328107	54.4	T/C	^∗∗∗∗∗∗∗^
	FS-2015-t8a	2015	t8	2.7	4.3	39.5	22.1	8_5646672	39.5	G/A	^∗∗∗∗∗∗^
	FS-2015-t8b	2015	t8	2.7	5.0	54.9	24.6	8_18164251	54.9	T/C	^∗∗∗∗∗∗∗^
	FS-2015-t9a	2015	t9	2.7	4.8	66.0	24.5	9_25649934	66.0	A/T	^∗∗∗∗∗∗∗^
	FS-2015-t9b	2015	t9	2.7	3.8	83.9	20.8	9_28915131	82.9	A/G	^∗∗∗∗∗∗∗^
	FS-2016-t6	2016	t6	2.7	5.5	47.8	29.9	6_20249564	49.7	A/G	^∗∗∗∗∗∗∗^
	FS-2016-t8	2016	t8	2.7	2.7	55.6	13.9	8_18728386	55.6	T/C	^∗∗∗∗^
	FS-2016-t9	2016	t9	2.7	3.2	83.9	18.1	9_29102006	84.9	C/T	^∗∗∗∗∗∗^
	FS-2015-s7a	2015	s7	2.6	4.7	56.3	25.3	7_11398231	56.3	A/T	^∗∗∗∗∗∗∗^
	FS-2015-s7b	2015	s7	2.6	5.5	67.1	29.1	7_15491756	67.1	T/C	^∗∗∗∗∗∗∗^
	FS-2016-s7a	2016	s7	2.6	3.5	43.9	19.5	7_7947819	43.9	C/T	^∗∗∗∗∗∗^
	FS-2016-s7b	2016	s7	2.6	3.1	73.5	17.3	8_20039431	73.5	G/A	^∗∗∗∗∗∗^
Canopy damage	CD-2015-t6	2015	t6	2.7	3.7	43.4	22.0	6_19837597	43.4	T/C	^∗∗∗∗∗∗∗^
	CD-2015-t8	2015	t8	2.7	3.0	50.2	16.8	8_8274031	50.2	C/G	^∗∗∗∗^
	CD-2015-t9	2015	t9	2.7	3.5	59.5	21.2	9_22115818	57.5	A/T	^∗∗∗∗∗∗^
	CD-2016-t6	2016	t6	2.7	2.8	27.9	14.6	6_17324675	27.9	C/A	^∗∗∗∗^
	CD-2016-t7	2016	t7	2.7	3.0	35.0	15.9	7_4403223	33.0	C/A	^∗∗∗∗∗^

*^a^The linkage group of trifoliate orange (t1–t9) or sweet orange (s1–s9) where QTL was detected. ^b^The threshold LOD score was determined by 1,000 permutation tests in the genome-wide map. ^c^The maximum LOD score of each QTL was determined by Interval Mapping. ^d^The QTL position (in cM) from the top of LG corresponding to the maximum LOD score. ^e^The percentage of the total phenotypic variation explained by QTL. ^f^The name of the nearest marker also represent the genomic position in the reference genome. ^g^The position (in cM) of the nearest marker from the top of the corresponding linkage group. ^h^The allele nucleobase type of the nearest marker with the first base unique in the detected parent. ^i^The significance level of the Kruskal–Wallis test: ^∗∗∗∗^p < 0.0001; ^∗∗∗∗∗^p < 0.00001; ^∗∗∗∗∗∗^p < 0.000001; ^∗∗∗∗∗∗∗^p < 0.0000001.*

**FIGURE 5:**
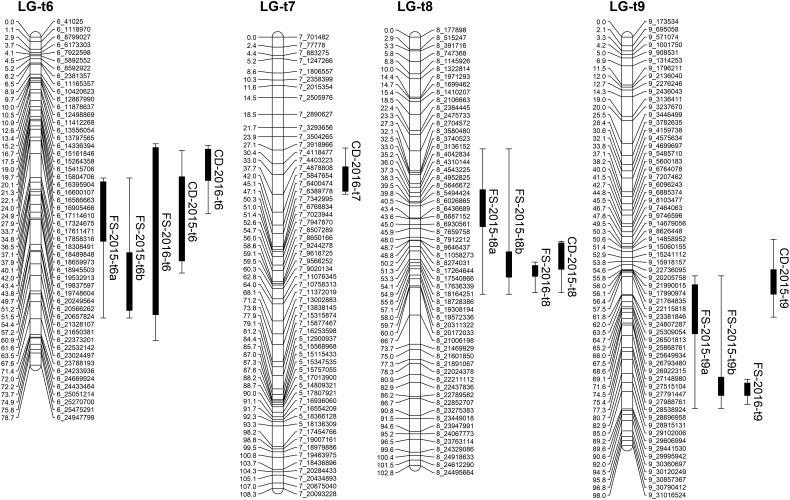
Mapping QTLs with foliar symptoms and canopy damage on the trifoliate orange genetic linkage map. Thick bars on the right side of each LG indicate confidence interval of QTLs with LOD score above threshold and flanking error bars indicate extension of QTL region at LOD score 2.0.

**FIGURE 6:**
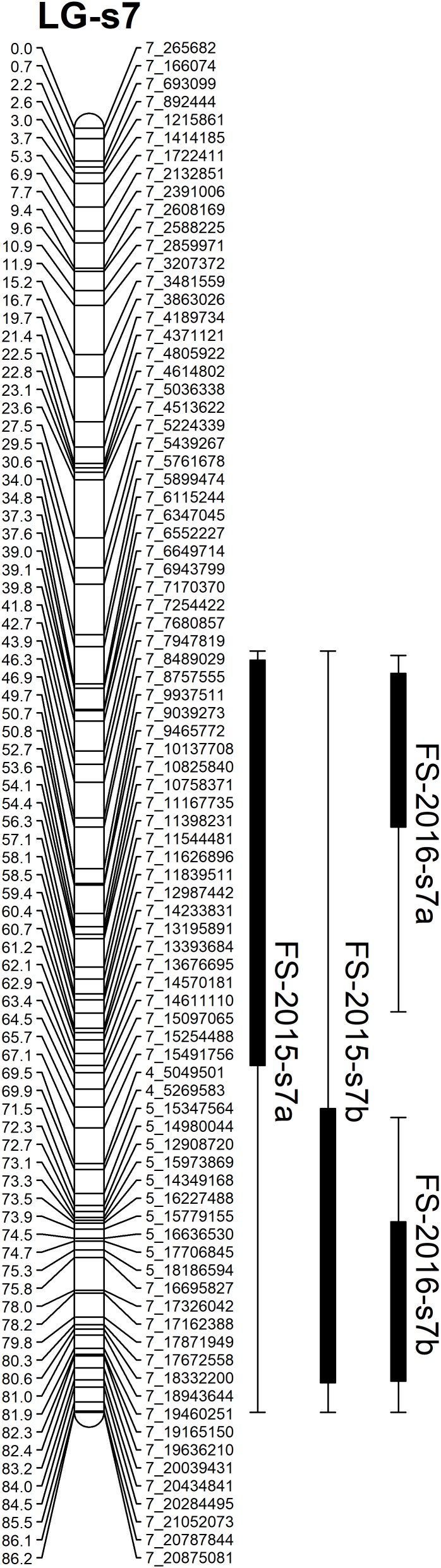
Mapping QTLs with foliar symptoms and canopy damage on the sweet orange genetic linkage map. Thick bars on the right side of each LG indicate confidence interval of QTLs with LOD score above threshold and flanking error bars indicate extension of QTL region at LOD score 2.0.

Based on the confidence intervals of the QTLs on the genetic maps, most of the QTLs are repeatable between the 2 years (Figures 5, 6). For the QTLs associated with foliar symptoms, seven QTLs were co-localized in three locations of the trifoliate orange map, on LG-t6, LG-t8 and LG-t9, while four QTLs were co-localized in two locations of LG-s7 on the sweet orange map. For the QTLs associated with canopy damage, only two QTLs on LG-t6 showed certain co-localization. It is noteworthy that co-localization of QTLs between the two traits were also observed in three locations of the trifoliate orange map, respectively, on LG-t6, LG-t8 and LG-t9. Overall, all the locations of QTLs associated with the two traits could be grouped into four main clusters, respectively, located on LG-t6, LG-t8, LG-t9 and LG-s7. The approximate genomic size of corresponding regions on the reference genome for these QTL clusters were Scaffold_6, Scaffold_8, Scaffold_9 and Scaffold_7. Among the QTL clusters, the one on LG-t6 was the most repeatable QTL and explained the largest part of the phenotypic variation.

## Discussion

### Genotyping and Linkage Mapping

In this study, we constructed two separate parental genetic maps, which, to our knowledge, are the highest density genetic maps to date for trifoliate orange and sweet orange. The former highest density genetic map of trifoliate orange consisted of 146 SNP markers and 74 SSR markers, spanning a total genetic length of 937.1 cM and having an average density of 0.23 marker/cM (Lyon, 2008). Our trifoliate orange map consisted of 647 unique-loci SNP markers with an average density of 0.63 marker/cM and had quite high coverage of the citrus genome. Thus, our genetic mapping effort has greatly improved the saturation of the genetic map of trifoliate orange. This new linkage map can be utilized as a reference map in trifoliate orange genome assembly in the future. For sweet orange, the previously reported highest density genetic map consisted of 799 SNP markers and 189 SSR markers spanning a total genetic length of 1026.6 cM with an average density of 0.95 marker/cM (Lyon, 2008). The map was used as a reference map for the assembly of a sweet orange genome (Xu et al., 2013). We achieved a slightly higher density in our genetic map (0.99 marker/cM), though the map consisted of less markers. It is probably attributed to better quality of the genotyping and linkage mapping, resulting in smaller size of the linkage groups for our sweet orange map.

However, citrus genetic maps require substantial additional effort to achieve the high resolution found in model species genetic maps, which include thousands of markers with high accuracy and precision. Although we initially developed thousands of SNP markers for our linkage mapping, only a small portion of these markers were successfully mapped as unique loci. This has been observed in all previous SNP-based genetic maps of citrus, where many markers were located in “zero recombination clusters”, indicating nearly no meiotic recombination occurs between markers within those regions (Lyon, 2008; Shimada et al., 2014; Guo et al., 2015; Yu et al., 2016; Curtolo et al., 2017; Imai et al., 2017). As shown in Figures 3 and 4, many markers in some small regions (<2 cM) of the linkage groups fell into clusters, which all correspond to very large genomic regions (5–10 Mb) on citrus chromosomes. High redundancy of markers is commonly attributed to small population size, closely adjacent physical marker location, and regions with low recombination, which could be significantly improved by increasing the size of mapping populations and the evenness of marker distribution. These solutions have been applied to mapping of many plant species but are difficult for citrus species. Biological characteristics of citrus, such as long juvenility, seedlessness, polyembryony, apomixis, heterozygosis, gametophytic incompatibility, zygotic selection and gametic selection (Shimada et al., 2014), not only seriously hamper the development of numerous uniquely segregating markers and production of large full-sib populations, but also remarkably influence allelic segregation and recombination ratio (Bernet et al., 2010; Ollitrault et al., 2012a). In citrus hybridizations, parental genotype and homology have obvious effects on genetic map density. Guo et al. (2015) successfully constructed an integrated genetic map of pummelo with 1543 SNP markers using an intraspecific full-sib F_1_ population with only 124 individuals. By contrast, Curtolo et al. (2017) attempted to construct a genetic map using an interspecific full-sib F_1_ population with 278 individuals from crossing of tangor and sweet orange, but only 661 non-redundant SNP-based DArTseq markers were finally mapped on the integrated map. The differences indicate that the use of intraspecific populations tends to achieve much higher marker density in the construction of genetic maps than interspecific populations, even though the population size is much smaller. In our study, the mapping population is neither interspecific nor intraspecific hybrids, but intergeneric hybrids, which had made it more difficult to achieve high-density genetic maps. The potential karyotypic and genomic divergences between trifoliate orange and sweet orange can prevent normal meiotic pairing and homology recombination during meiosis. Some genomic divergences between the two species can be obviously observed from the conservation of synteny of the genetic linkage maps and the reference genome, e.g., the divergences on LG-4, LG-7 and LG-8. In addition to parental genotype, differential fitness of gamete genotypes, crossing direction, and regulatory gene interactions likely also contribute to the high level of segregation distortion in citrus (Bernet et al., 2010; Curtolo et al., 2017). It should be noted that our mapping population was not a full-sib family, but mixed progenies from three different crosses between two trifoliate orange varieties and two sweet orange varieties. Our genotyping results show that only 3.4 and 1.8% of genotyped SNP loci are inconsistent, respectively, between the trifoliate oranges and sweet oranges. However, when compared to the identified segregating SNP markers, the percentages rise up to 29.6 and 6.1%, respectively (Supplementary File S2). This is a substantial percentage of inconsistent segregating markers in trifoliate orange. By analyzing the distribution of these inconsistent SNP loci in trifoliate orange on the reference genome, we found that they are mostly concentrated in certain regions of the genome, and the two trifoliate oranges almost share all of these regions. Therefore, elimination of these polymorphic loci not only reduced the number of available segregating SNP markers, especially for the trifoliate orange map, but also resulted in several large regions with fewer markers on the genetic maps. Actually, many large gaps on the genetic maps were rightly located in the positions of the eliminated polymorphic SNP loci. In addition, in comparison to sweet orange, many fewer segregating markers were identified in trifoliate orange (Supplementary File S2). This problem was reported in all previous genetic maps of trifoliate orange (Chen et al., 2008; Lyon, 2008; Ollitrault et al., 2012a). The low availability of segregating markers in trifoliate orange is due to its lower heterozygosity. As the current sequence coverage only accounts for 2.3% of the citrus genome, to maximize detection of segregating SNP markers, greater sequence coverage is needed for the progenies and parents.

Significant differences in linkage group sizes were observed between trifoliate orange and sweet orange genetic maps. Each of the linkage groups of trifoliate orange is larger in genetic length than the corresponding one of sweet orange, even though there were less markers mapped on the trifoliate orange genetic map. Similar differences in genetic distances were evident in the previously reported EST-SSR genetic maps based on codominant markers segregating in both parents (Chen et al., 2008). Variation in map length could also be observed between SNP-based genetic maps of mandarin, pummelo and sweet orange (Ollitrault et al., 2012a). Due to the utilization of a reference genome, SNP-based genetic linkage maps are comparable if referred to physical distance. The variation in genetic map length is unrelated to genome size among different citrus species, which range from 360 to 398 Mb (Gmitter et al., 2012; Wu et al., 2014, 2018). Extensively distributed in our maps, nearly half of the markers exhibited significant segregation distortion, and such segregation distortion in citrus was proposed to result from gametic selection rather than zygotic selection (Ollitrault et al., 2012a). However, in a simulation study on factors affecting linkage map construction, segregation distortion from gametic selection had little influence on marker order and genetic distance (Hackett and Broadfoot, 2003). Thus, variation of genetic size between linkage group maps of trifoliate orange and sweet orange probably is not due to segregation distortion, but reflects differential recombination rates between the species. Studies on model plants amply demonstrate the impact of genome sequence divergence on recombination rate, and lower recombination rate is related to higher levels of genome divergence (Chetelat et al., 2000; Opperman et al., 2004; Li L. et al., 2006). For different citrus-related genera and species, the degree of genome heterozygosity differs dramatically. Sweet orange, known to be highly heterozygous, was demonstrated to be a complex interspecific hybrid derived from pummelo and mandarin (Xu et al., 2013; Wu et al., 2014). However, trifoliate orange, a genus related to *Citrus*, was found to be lower in heterozygosity (Chen and Gmitter, 2013). Therefore, in comparison to trifoliate orange, higher heterozygosity in the sweet orange genome probably suppresses recombination frequency, resulting in a smaller genetic size. This is in agreement with the difference in genetic distance between shared markers on genetic maps of Clementine mandarin and pummelo (Ollitrault et al., 2012a). Clementine mandarin is an interspecific hybrid of *C. reticulata* × *C. sinensis* with high genome heterozygosity (Wu et al., 2014), while pummelo is a progenitor species in *Citrus* with low genome heterozygosity (Wang X. et al., 2017). In addition, recombination rates are known to differ between sexes in both plants and animals (Lorch, 2005). The size of the male genetic map of Clementine mandarin was notably larger than its female genetic map (Ollitrault et al., 2012a). Our mapping population was a mix of several crosses between different varieties of trifoliate orange and sweet orange with taxa serving as male and female parents in different crosses. However, most of the progenies were generated with trifoliate orange as the male parent, which may also contribute to the larger size of the trifoliate orange genetic map.

### Phenotyping and QTL Mapping

This is the first report on identification of QTLs related to HLB disease and tolerance. Most of the recently reported QTLs in citrus are related to morphological and physiological traits (Kepiro and Roose, 2010; Sugiyama et al., 2011; Sahin-Cevik and Moore, 2012; Raga et al., 2014; Asins et al., 2015; Raga et al., 2016; Yu et al., 2016; Curtolo et al., 2017; Imai et al., 2017; Yu et al., 2017). Only a few reports focused on disease-related QTLs in citrus, such as resistance to citrus tristeza virus (CTV) (Asins et al., 2012; Ohta et al., 2015), Alternaria brown spot (ABS) (Cuenca et al., 2013, 2016), citrus leprosis virus (CiLV) (Bastianel et al., 2009), and citrus nematode (Ling et al., 2000). In genetic studies, estimated effects of each QTL for disease resistance in plants usually ranges from a few percent to 50% or more, and a QTL accounting for phenotypic variation of more than 20% is commonly described as a major QTL, or more than 50% as a dominant QTL (Davey et al., 2006; St Clair, 2010). Interestingly, only one QTL was found with a dominant effect on the phenotypic resistance for each of the diseases, suggesting that the inheritance of resistance for these diseases is mainly controlled by a single dominant allele. In our study, four clusters of QTLs associated with HLB tolerance were identified on the two parental genetic maps. Although all of them could be considered as major QTLs, none of them alone could explain a majority of the phenotypic variation. Our results indicate that the high degree of tolerance to HLB in trifoliate orange cannot be monogenic, because at least four genomic regions are involved. However, the HLB pathogenesis mechanism is still unknown and is likely complicated (Martinelli and Dandekar, 2017;Wang N.A. et al., 2017). Based on the most recent progress in studies on HLB, it was proposed that at least three main molecular mechanisms occur in citrus in response to HLB, and these mechanisms involve many pathways and genes (Martinelli and Dandekar, 2017). This is in agreement with our results that citrus tolerance to HLB is polygenic.

Unlike CTV, CiLV, ABS and citrus nematode for which strong resistance is available within the citrus gene pool, the suppression of HLB in trifoliate orange may be best described as tolerance. Trifoliate orange had been reported as resistant to *C*Las infection (Folimonova et al., 2009), but recently more studies suggested that it is not true resistant but highly tolerant (Ramadugu et al., 2016; Miles et al., 2017). In our study, though the trifoliate oranges were generally determined to be HLB-negative, most of the replicate trees had marginal results for *C*Las diagnosis (C*t* value of qPCR ranging from 33 to 39), and a few trees were even HLB-positive (C*t* value of qPCR under 33). It is important to note that at least eight replicate trees were clonally propagated for each genotype and they all were used throughout the phenotyping, which ensured high accuracy and reliability of the phenotypic results for each genotype. Thus, we believed that the trifoliate orange was probably infected by *C*Las, but the *C*Las titer was held at relatively low levels. Our results on HLB disease evaluation also support this interpretation. The trifoliate oranges were not completely healthy under the intense HLB pressure, but mostly displayed slight foliar disease symptoms and slight canopy damage. The results further suggest that trifoliate oranges are not immune to *C*Las infection, but can inhibit growth of *C*Las and show low symptom levels when they are infected. It is noteworthy that obviously different degrees of tolerance to HLB was observed among the F_1_ progenies, and a few progenies showed similar results of *C*Las diagnosis and disease evaluation as trifoliate oranges, indicating the HLB tolerance is inheritable. Moreover, some other hybrids containing the germplasm of trifoliate orange were also found with certain tolerance to HLB (Albrecht and Bowman, 2011, 2012; Ramadugu et al., 2016), probably due to the partial inheritances of HLB tolerance from trifoliate orange.

Although high-density genetic maps were constructed for both trifoliate orange and sweet orange, the outcome of QTL mapping was still not fine enough for anchoring specific genomic regions or specifying candidate genes associated with citrus responses to HLB. The four anchored genomic regions spanned a total genomic length of approximately 44.6 Mb, consisting of thousands of genes. These genomic regions involved 57 markers from three regions of the trifoliate orange map and 54 markers from one region of the sweet orange map, indicating the marker density of our genetic maps is sufficient for fine mapping. One possible explanation for such phenomenon could be that the quality of the constructed genetic maps is still insufficient for fine mapping of QTLs. The characteristics of plant materials themselves and the utilization of mixed intergeneric populations for genotyping likewise may restrict the construction of genetic maps with sufficient resolution and accuracy. All the linkage groups in trifoliate orange where the QTLs were detected were constructed with fewer markers, especially within the regions of some QTLs. Another possible explanation could be that the size of the phenotyping population is not large enough to yield fine phenotypic data with wide variation. Due to the space requirements for trees and test costs, only 86 of the progenies were selected for phenotyping in our study, accounting for only a half of the genotyping population. Moreover, it is also possible that each of these regions consist of multiple QTLs and they generally co-segregated in the population. As presented in the graphs of QTLs, many QTLs have more than one peak within the LOD score profile (Supplementary Figure S2). Increasing the size of the phenotyping population, to equal that of the genotyping population, should greatly improve the quality of QTL mapping and minimize the number of anchored genomic regions associated with HLB tolerance; however, such larger experiments with tree crops can become cost-prohibitive, in comparison with similar experimental designs with annual or model plant species.

## Conclusion

Based on Genotyping-by-Sequencing of an intergeneric F_1_ population of 170 progenies, we constructed two high-density genome-wide genetic maps for trifoliate orange and sweet orange. Each of the genetic maps contained nine firm linkage groups corresponding to the haploid chromosome number of the species and exhibited high synteny and high coverage of the reported citrus genome. The minor discrepancies among the genetic maps and genome assemblies may represent possible structural rearrangements among citrus species, or alternatively errors in the previous genome assembly. In the replicated field evaluation over 2 years, trifoliate orange and sweet orange showed significant differences in response to *C*Las infection and HLB, and their progenies exhibited an obvious continuous distribution for two phenotypic traits. Four clusters of QTLs were identified for HLB-incited foliar and canopy responses, respectively, located on LG-t6, LG-t8 and LG-t9 of the trifoliate orange genetic map and LG-s7 of the sweet orange genetic map. These QTLs collectively explained a major part of the phenotypic variation in response to HLB disease. Our results suggest that multiple QTLs are involved in the genetic control of HLB response in citrus. This work provides a starting point for future studies of the underlying genetic architecture of resistance or tolerance to HLB. These QTLs need to be confirmed further to facilitate breeding for resistance or tolerance to HLB in citrus. In addition, the corresponding genomic regions need to be refined if the objective is to discover and characterize candidate genes related to the host response to disease. The final identified QTLs and genes could be good targets for citrus breeding to support long-term solutions to this devastating disease.

## Author Contributions

FG, ES, MR, ZD, and MH conceived the study. FG and MR developed the mapping population. MR, MH, and QY conducted the work of genotyping. MH, QY, DD, YY, and YZ conducted the work of phenotyping. MH and MR analyzed the genotypic data. MH analyzed the phenotypic data, performed the QTL mapping, and drafted the manuscript. All authors read and approved the final manuscript.

## Conflict of Interest Statement

The authors declare that the research was conducted in the absence of any commercial or financial relationships that could be construed as a potential conflict of interest. The handling Editor declared a shared affiliation, though no other collaboration, with several of the authors MH, QY, DD, YY, YZ, and FG.

## Footnotes

1 http://www.floridacitrus.org

2 https://phytozome.jgi.doe.gov

## Supplementary Material

FIGURE S1 Collinear analysis of consensus between genetic linkage maps and reference genome by dot-plot diagram.

FIGURE S2 Full genetic linkage maps and genome-wide LOD score graphics of QTL mapping of two phenotypic traits.

FILE S1 Summary of Genotyping by Sequencing for 170 F_1_ progenies and four parental varieties.

FILE S2 Analysis of parental genotypes and distribution of SNP loci on reference genome in four parental varieties.

FILE S3 Diagnosis of *C*Las infection by real-time qPCR in 86 F_1_ progenies and nine control varieties.

FILE S4 Phenotypic data of two traits for the evaluation of HLB disease in 86 F_1_ progenies and nine control varieties.
