# Supplementary figures and images for: Construction of High-Density Genetic Maps and Detection of QTLs Associated With Huanglongbing Tolerance in Citrus
Source: Front Plant Sci. 2018 Nov 27;9:1694. doi: 10.3389/fpls.2018.01694 (PMC6278636; doi:10.3389/fpls.2018.01694)

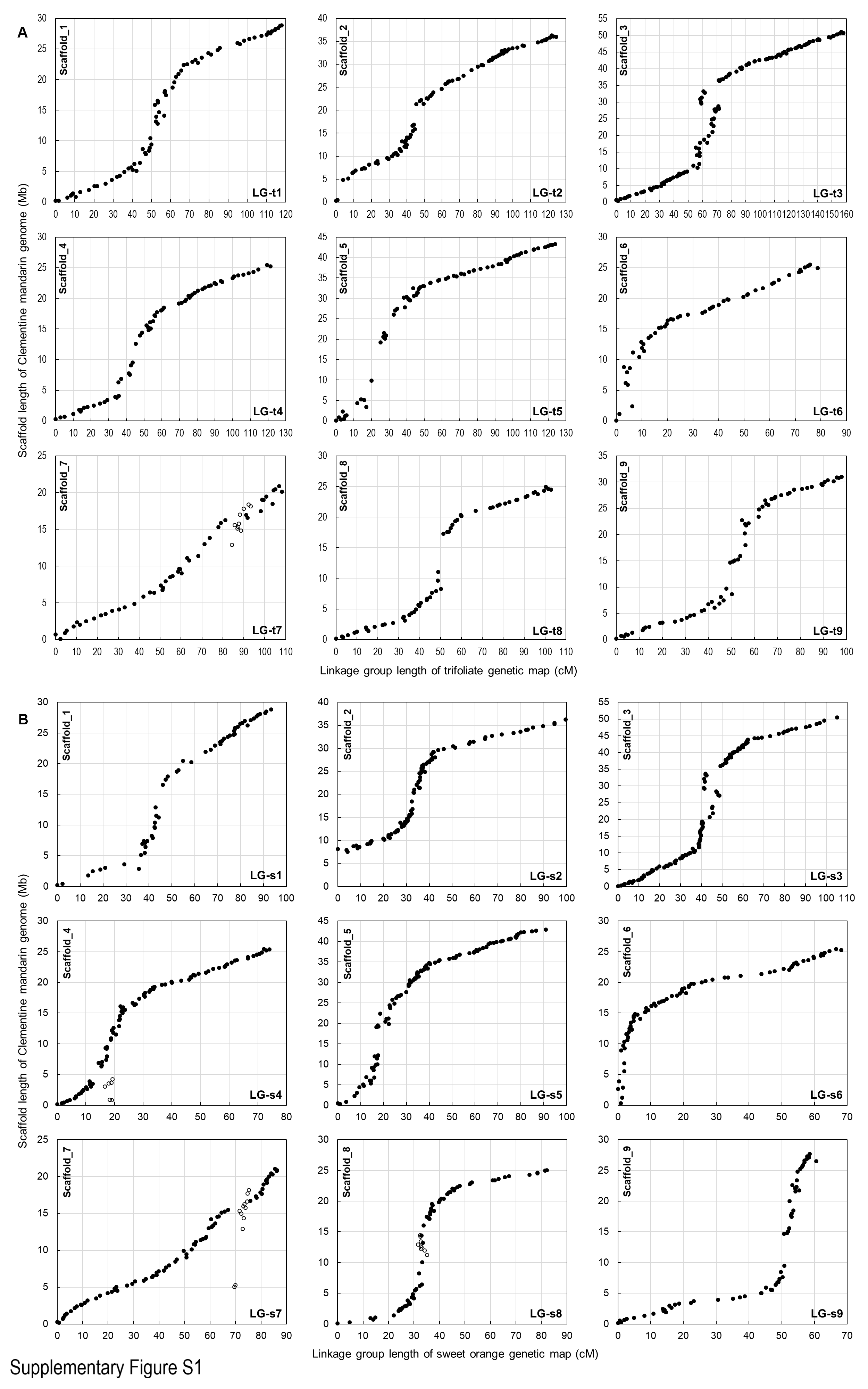

Supplement: FIGURE S1 — Collinear analysis of consensus between genetic linkage maps and reference genome by dot-plot diagram. [file Image_1.tif]

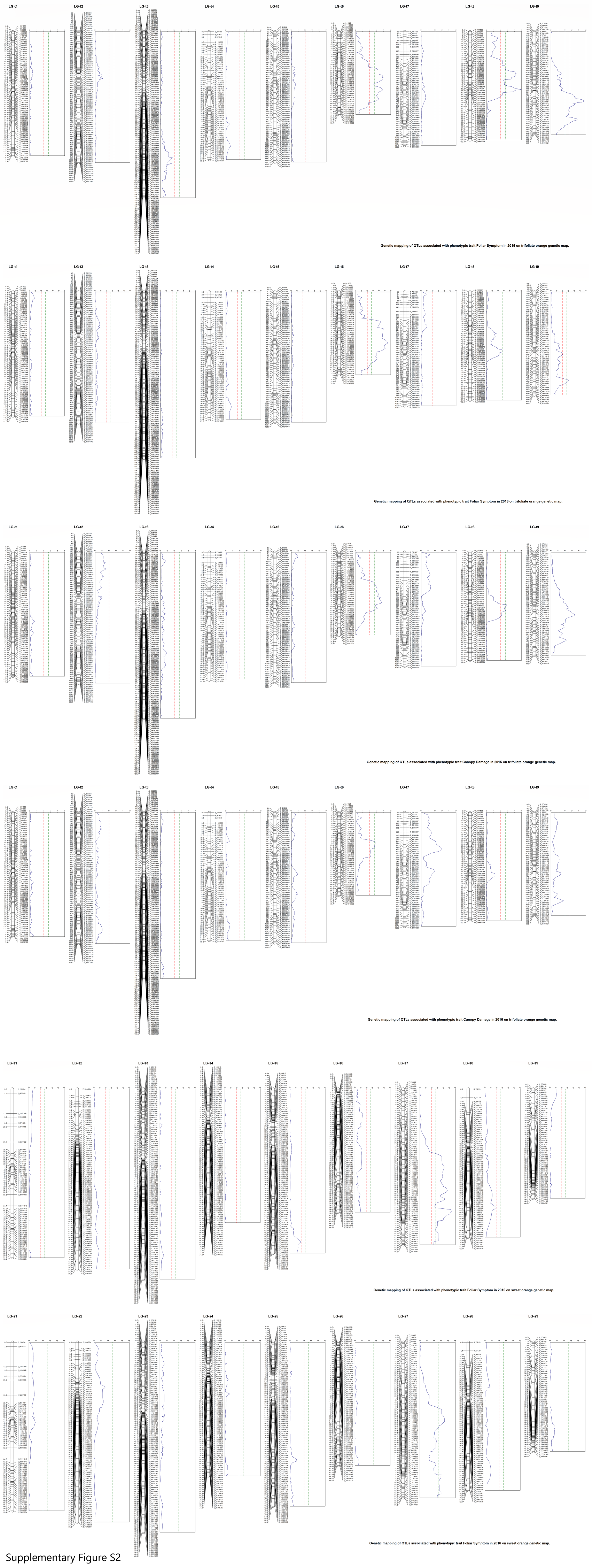

Supplement: FIGURE S2 — Full genetic linkage maps and genome-wide LOD score graphics of QTL mapping of two phenotypic traits. [file Image_2.tif]
