# Supplementary material for: Construction of High-Density Genetic Maps and Detection of QTLs Associated With Huanglongbing Tolerance in Citrus
Source: Front Plant Sci. 2018 Nov 27;9:1694. doi: 10.3389/fpls.2018.01694 (PMC6278636; doi:10.3389/fpls.2018.01694)
Supplement: FILE S1 — Summary of Genotyping by Sequencing for 170 F1 progenies and four parental varieties. [file Table_1.docx]

**Supplementary File S1** Summary of Genotyping by Sequencing for 170 F_1_ progenies and four parental varieties

| SampleCode | Cross type | ReadCount | Clusters | Med_Depth |
| --- | --- | --- | --- | --- |
| 13 | FDTxSuc | 6055667 | 93794 | 34 |
| 14 | FDTxSuc | 3150765 | 100944 | 17 |
| 15 | FDTxSuc | 2941236 | 93985 | 17 |
| 16 | FDTxSuc | 2070282 | 79536 | 14 |
| 17 | FDTxSuc | 4110949 | 108440 | 21 |
| 18 | FDTxSuc | 4217099 | 109346 | 22 |
| 19 | FDTxSuc | 3321681 | 96790 | 18 |
| 20 | FDTxSuc | 2087912 | 48201 | 23 |
| 21 | FDTxSuc | 2936813 | 59657 | 26 |
| 22 | FDTxSuc | 1518980 | 37917 | 22 |
| 23 | FDTxSuc | 1293865 | 33460 | 22 |
| 24 | FDTxSuc | 4920208 | 83852 | 31 |
| 25 | FDTxSuc | 4029600 | 74815 | 28 |
| 26 | FDTxSuc | 1989710 | 81558 | 14 |
| 27 | FDTxSuc | 4516668 | 112667 | 23 |
| 28 | FDTxSuc | 2999273 | 97405 | 17 |
| 29 | FDTxSuc | 6547205 | 122541 | 33 |
| 30 | FDTxSuc | 4317203 | 112473 | 21 |
| 31 | FDTxSuc | 2893435 | 90117 | 16 |
| 32 | FDTxSuc | 1962043 | 47539 | 23 |
| 33 | FDTxSuc | 2299371 | 54325 | 23 |
| 34 | FDTxSuc | 2340660 | 54945 | 24 |
| 35 | FDTxSuc | 2815810 | 56927 | 25 |
| 36 | FDTxSuc | 4720384 | 83404 | 30 |
| 42 | FDTxSuc | 6988131 | 110066 | 33 |
| 47 | FDTxSuc | 3093339 | 94023 | 16 |
| 48 | FDTxSuc | 2784528 | 94075 | 16 |
| 49 | FDTxSuc | 3602964 | 100404 | 18 |
| 50 | FDTxSuc | 1220535 | 31477 | 21 |
| 51 | FDTxSuc | 2709885 | 58009 | 25 |
| 173 | FDTxSuc | 2445763 | 53272 | 24 |
| 174 | FDTxSuc | 1800141 | 45473 | 22 |
| 175 | FDTxSuc | 3121392 | 62158 | 26 |
| 176 | FDTxSuc | 2002338 | 77910 | 13 |
| 177 | FDTxSuc | 2476962 | 58374 | 23 |
| 185 | FDTxSuc | 3680555 | 102945 | 20 |
| 186 | FDTxSuc | 4508115 | 76117 | 30 |
| 187 | FDTxSuc | 1507866 | 35290 | 23 |
| 188 | FDTxSuc | 3344808 | 70057 | 27 |
| 189 | FDTxSuc | 3835254 | 110953 | 20 |
| 190 | FDTxSuc | 4609763 | 115882 | 23 |
| 207 | FDTxSuc | 3472764 | 107458 | 18 |
| 208 | FDTxSuc | 6546758 | 125122 | 29 |
| 209 | FDTxSuc | 6943750 | 132968 | 27 |
| 212 | FDTxSuc | 6503050 | 94771 | 32 |
| 214 | FDTxSuc | 8234309 | 106761 | 38 |
| 215 | FDTxSuc | 4436870 | 86318 | 30 |
| 217 | FDTxSuc | 5415714 | 69665 | 36 |
| 278 | FDTxSuc | 5369893 | 89887 | 31 |
| 279 | FDTxSuc | 7308704 | 102402 | 38 |
| 280 | FDTxSuc | 6278970 | 94467 | 33 |
| 111 | SanxATf | 10485016 | 118188 | 43 |
| 117 | SanxATf | 1263892 | 63651 | 9 |
| 118 | SanxATf | 2097539 | 94287 | 11 |
| 119 | SanxATf | 2279889 | 100892 | 11 |
| 122 | SanxATf | 2777116 | 96685 | 13 |
| 123 | SanxATf | 1643640 | 66368 | 11 |
| 124 | SanxATf | 1491116 | 65791 | 10 |
| 126 | SanxATf | 3540153 | 139499 | 11 |
| 127 | SanxATf | 3014899 | 100696 | 15 |
| 128 | SanxATf | 2035790 | 84190 | 12 |
| 130 | SanxATf | 2238536 | 86516 | 12 |
| 132 | SanxATf | 1266920 | 59223 | 9 |
| 133 | SanxATf | 1138805 | 52947 | 9 |
| 136 | SanxATf | 11380336 | 207194 | 22 |
| 137 | SanxATf | 2742735 | 98953 | 13 |
| 141 | SanxATf | 2002082 | 67714 | 12 |
| 143 | SanxATf | 1864342 | 64181 | 11 |
| 144 | SanxATf | 571704 | 20836 | 9 |
| 146 | SanxATf | 2263804 | 103479 | 10 |
| 149 | SanxATf | 1833443 | 70335 | 12 |
| 152 | SanxATf | 2058724 | 72044 | 12 |
| 154 | SanxATf | 2733778 | 80690 | 13 |
| 155 | SanxATf | 8917888 | 168923 | 21 |
| 156 | SanxATf | 2392906 | 83724 | 12 |
| 158 | SanxATf | 1930927 | 62484 | 12 |
| 162 | SanxATf | 2577181 | 92705 | 12 |
| 163 | SanxATf | 2832969 | 97154 | 14 |
| 164 | SanxATf | 1338246 | 66332 | 10 |
| 166 | SanxATf | 7253189 | 181375 | 17 |
| 167 | SanxATf | 1513609 | 71280 | 10 |
| 168 | SanxATf | 1056087 | 43893 | 10 |
| 169 | SanxATf | 20649146 | 278832 | 13 |
| 170 | SanxATf | 4143833 | 109245 | 16 |
| 178 | SanxATf | 1053217 | 37709 | 10 |
| 179 | SanxATf | 8826353 | 170141 | 24 |
| 180 | SanxATf | 14914372 | 240277 | 19 |
| 181 | SanxATf | 7336699 | 161323 | 23 |
| 182 | SanxATf | 2156782 | 89122 | 12 |
| 183 | SanxATf | 2402836 | 92327 | 11 |
| 184 | SanxATf | 1669818 | 81427 | 10 |
| 193 | SanxATf | 3833984 | 134005 | 14 |
| 194 | SanxATf | 4964617 | 156301 | 14 |
| 195 | SanxATf | 4811625 | 146320 | 15 |
| 196 | SanxATf | 4369204 | 137789 | 15 |
| 197 | SanxATf | 4987344 | 143224 | 16 |
| 231 | SanxATf | 4093263 | 117607 | 16 |
| 232 | SanxATf | 4552902 | 126862 | 19 |
| 233 | SanxATf | 7409471 | 99927 | 35 |
| 234 | SanxATf | 3701332 | 105479 | 18 |
| 235 | SanxATf | 7446693 | 131175 | 30 |
| 236 | SanxATf | 2173718 | 85339 | 12 |
| 237 | SanxATf | 2286914 | 81566 | 12 |
| 238 | SanxATf | 3145379 | 105042 | 14 |
| 239 | SanxATf | 12062718 | 204810 | 23 |
| 240 | SanxATf | 1824254 | 79571 | 12 |
| 241 | SanxATf | 4930866 | 120231 | 17 |
| 242 | SanxATf | 4891400 | 125740 | 18 |
| 248 | SanxATf | 2368569 | 88304 | 12 |
| 249 | SanxATf | 4531078 | 118358 | 16 |
| 250 | SanxATf | 2934716 | 99698 | 13 |
| 251 | SanxATf | 3192230 | 97543 | 13 |
| 252 | SanxATf | 5301172 | 137032 | 17 |
| 253 | SanxATf | 3344793 | 107913 | 13 |
| 254 | SanxATf | 2542813 | 86119 | 13 |
| 256 | SanxATf | 4852152 | 125925 | 17 |
| 257 | SanxATf | 5249114 | 130921 | 18 |
| 258 | SanxATf | 4304829 | 120105 | 17 |
| 259 | SanxATf | 2960517 | 102981 | 14 |
| 261 | SanxATf | 4154842 | 123025 | 15 |
| 262 | SanxATf | 2203540 | 92919 | 12 |
| 263 | SanxATf | 1825068 | 78547 | 11 |
| 264 | SanxATf | 3260222 | 110620 | 15 |
| 265 | SanxATf | 4214673 | 116493 | 17 |
| 266 | SanxATf | 4200744 | 120840 | 18 |
| 270 | SanxATf | 2928239 | 102321 | 14 |
| 284 | SanxATf | 5035387 | 130329 | 20 |
| 286 | SanxATf | 3704894 | 123539 | 16 |
| 287 | SanxATf | 4574345 | 126874 | 19 |
| 271 | SanxATf | 7885082 | 130620 | 38 |
| 55 | SucxFDT | 3235991 | 100063 | 17 |
| 56 | SucxFDT | 1840057 | 78130 | 13 |
| 58 | SucxFDT | 1897033 | 73668 | 13 |
| 59 | SucxFDT | 6163532 | 125424 | 29 |
| 61 | SucxFDT | 2586946 | 54986 | 23 |
| 62 | SucxFDT | 5798875 | 93664 | 33 |
| 63 | SucxFDT | 3434529 | 70789 | 26 |
| 64 | SucxFDT | 3186404 | 62354 | 23 |
| 65 | SucxFDT | 2777820 | 92840 | 15 |
| 66 | SucxFDT | 2591907 | 86835 | 15 |
| 67 | SucxFDT | 2847805 | 91697 | 16 |
| 68 | SucxFDT | 3652383 | 105873 | 20 |
| 69 | SucxFDT | 3349253 | 98682 | 17 |
| 70 | SucxFDT | 3054265 | 64504 | 24 |
| 71 | SucxFDT | 13095632 | 126587 | 49 |
| 72 | SucxFDT | 2843852 | 57631 | 25 |
| 73 | SucxFDT | 3315168 | 62933 | 25 |
| 75 | SucxFDT | 3712229 | 111303 | 14 |
| 76 | SucxFDT | 5114261 | 132335 | 19 |
| 77 | SucxFDT | 4134282 | 121761 | 16 |
| 78 | SucxFDT | 5599617 | 141140 | 19 |
| 79 | SucxFDT | 1465577 | 66958 | 10 |
| 80 | SucxFDT | 3378854 | 108820 | 15 |
| 81 | SucxFDT | 1620944 | 63285 | 11 |
| 82 | SucxFDT | 2064787 | 71339 | 12 |
| 85 | SucxFDT | 3521571 | 113970 | 16 |
| 191 | SucxFDT | 3983625 | 128496 | 17 |
| 192 | SucxFDT | 3886635 | 120776 | 17 |
| 243 | SucxFDT | 5124065 | 87181 | 30 |
| 244 | SucxFDT | 7998662 | 144953 | 29 |
| 246 | SucxFDT | 4502860 | 76723 | 29 |
| 247 | SucxFDT | 4965703 | 124918 | 17 |
| 272 | SucxFDT | 5714020 | 137158 | 20 |
| 273 | SucxFDT | 4597546 | 126096 | 18 |
| 274 | SucxFDT | 4890382 | 130216 | 18 |
| 275 | SucxFDT | 5035207 | 128143 | 19 |
| 276 | SucxFDT | 4859983 | 127963 | 18 |
| 277 | SucxFDT | 4500501 | 124064 | 18 |
| 285 | SucxFDT | 8660731 | 106883 | 39 |
| 288 | SucxFDT | 4476660 | 74216 | 32 |
| 171 | Argentina trifoliate | 11342897 | 261282 | 9 |
| 198 | Flying Dragon trifoliate | 4943925 | 105090 | 20 |
| 199 | Succari sweet orange | 6092943 | 136610 | 21 |
| 269 | Sanford sweet orange | 8638464 | 156452 | 23 |
