# Supplementary material for: Construction of High-Density Genetic Maps and Detection of QTLs Associated With Huanglongbing Tolerance in Citrus
Source: Front Plant Sci. 2018 Nov 27;9:1694. doi: 10.3389/fpls.2018.01694 (PMC6278636; doi:10.3389/fpls.2018.01694)
Supplement: FILE S3 — Diagnosis of CLas infection by real-time qPCR in 86 F1 progenies and nine control varieties. [file Table_3.docx]

**Supplementary File S3** Diagnosis of *C*Las infection by real-time qPCR in 86 F_1_ progenies and nine control varieties. The Ct values for the diagnosis of *C*Las genome in citrus leaves were detected by the TaqMan label-based real-time qPCR. Trees with Ct value under 33 were considered to be HLB-positive. Each individual have eight replicate trees.

| Ind code | Cross | Ct 2015 |  | Ct 2016 |  | Percent 2015 | Percent 2016 |
| --- | --- | --- | --- | --- | --- | --- | --- |
|  |  | Mean | SD | Mean | SD | % | % |
| 1 | SanxATf | 26.1 | 2.4 | 27.4 | 2.2 | 83.3 | 91.7 |
| 2 | SanxATf | 25.8 | 2.3 | 26.8 | 1.3 | 79.2 | 100.0 |
| 3 | SanxATf | 27.8 | 4.8 | 28.0 | 1.9 | 62.5 | 95.8 |
| 4 | SanxATf | 28.0 | 3.3 | 27.8 | 1.6 | 56.5 | 95.8 |
| 5 | SanxATf | 29.8 | 5.1 | 29.0 | 3.0 | 58.3 | 83.3 |
| 6 | FDTxSuc | 27.3 | 3.7 | 28.6 | 0.9 | 79.2 | 91.7 |
| 7 | SucxFDT | 25.6 | 2.5 | 26.8 | 0.5 | 91.7 | 100.0 |
| 8 | FDTxSuc | 33.5 | 5.6 | 30.8 | 3.1 | 47.6 | 76.5 |
| 9 | SucxFDT | 25.1 | 1.6 | 27.0 | 0.6 | 100.0 | 100.0 |
| 10 | SanxATf | 25.7 | 1.5 | 26.8 | 0.8 | 91.7 | 100.0 |
| 11 | SanxATf | 28.1 | 4.6 | 27.9 | 0.6 | 70.8 | 100.0 |
| 12 | SanxATf | 28.6 | 4.1 | 29.1 | 2.2 | 79.2 | 83.3 |
| 13 | SanxATf | 25.2 | 2.8 | 26.6 | 2.1 | 91.7 | 95.8 |
| 14 | SucxFDT | 29.2 | 5.1 | 30.5 | 3.7 | 75.0 | 79.2 |
| 15 | FDTxSuc | 26.6 | 3.1 | 28.3 | 2.4 | 87.5 | 95.8 |
| 16 | FDTxSuc | 29.9 | 6.5 | 31.0 | 3.0 | 52.2 | 71.4 |
| 17 | SanxATf | 29.0 | 5.7 | 28.8 | 2.4 | 62.5 | 83.3 |
| 18 | SanxATf | 27.3 | 3.2 | 26.7 | 1.1 | 58.3 | 100.0 |
| 19 | FDTxSuc | 27.0 | 2.5 | 28.6 | 1.4 | 87.5 | 95.8 |
| 20 | SucxFDT | 27.8 | 3.6 | 27.5 | 1.2 | 83.3 | 95.8 |
| 21 | FDTxSuc | 32.4 | 6.0 | 28.4 | 1.8 | 56.5 | 83.3 |
| 22 | SucxFDT | 28.3 | 4.2 | 28.4 | 2.4 | 70.8 | 83.3 |
| 23 | SanxATf | 26.4 | 2.9 | 27.4 | 1.9 | 83.3 | 95.8 |
| 24 | SanxATf | 26.0 | 2.2 | 27.2 | 1.0 | 83.3 | 95.8 |
| 25 | SucxFDT | 27.6 | 3.3 | 27.1 | 0.8 | 79.2 | 100.0 |
| 26 | SanxATf | 27.8 | 3.2 | 28.8 | 2.0 | 70.8 | 91.7 |
| 27 | SanxATf | 28.3 | 4.9 | 27.9 | 0.7 | 79.2 | 100.0 |
| 28 | FDTxSuc | 28.3 | 5.5 | 27.4 | 1.4 | 83.3 | 100.0 |
| 29 | FDTxSuc | 33.7 | 6.2 | 33.4 | 4.8 | 29.2 | 52.4 |
| 30 | SanxATf | 29.9 | 5.4 | 28.9 | 1.5 | 62.5 | 95.8 |
| 31 | SanxATf | 26.2 | 1.7 | 28.2 | 2.1 | 87.5 | 87.5 |
| 32 | SucxFDT | 29.3 | 1.9 | 29.1 | 0.4 | 71.4 | 100.0 |
| 33 | SucxFDT | 28.9 | 5.9 | 27.6 | 1.1 | 75.0 | 100.0 |
| 34 | SanxATf | 25.3 | 1.0 | 27.3 | 2.2 | 87.5 | 95.8 |
| 35 | FDTxSuc | 31.2 | 3.4 | 30.1 | 1.9 | 43.5 | 66.7 |
| 36 | FDTxSuc | 26.2 | 1.7 | 30.2 | 2.0 | 83.3 | 87.5 |
| 37 | FDTxSuc | 25.8 | 2.8 | 27.9 | 1.1 | 79.2 | 95.8 |
| 38 | SucxFDT | 27.4 | 4.3 | 27.5 | 2.2 | 75.0 | 91.7 |
| 39 | SanxATf | 26.6 | 3.6 | 27.3 | 1.8 | 83.3 | 91.7 |
| 40 | SanxATf | 26.6 | 2.8 | 28.7 | 1.8 | 87.5 | 91.7 |
| 41 | SanxATf | 30.3 | 4.1 | 29.6 | 2.8 | 58.3 | 87.5 |
| 42 | SanxATf | 27.6 | 1.6 | 28.9 | 2.1 | 79.2 | 87.5 |
| 43 | FDTxSuc | 29.0 | 2.2 | 29.1 | 2.5 | 62.5 | 87.5 |
| 44 | SanxATf | 26.8 | 2.5 | 28.7 | 1.5 | 75.0 | 91.7 |
| 45 | SucxFDT | 28.3 | 2.9 | 29.2 | 2.1 | 70.8 | 87.5 |
| 46 | SanxATf | 27.1 | 2.3 | 27.8 | 1.3 | 79.2 | 95.8 |
| 47 | SanxATf | 25.5 | 1.6 | 28.3 | 1.9 | 87.5 | 91.7 |
| 48 | SanxATf | 28.5 | 3.8 | 28.4 | 1.9 | 62.5 | 95.8 |
| 49 | SanxATf | 28.7 | 3.0 | 27.4 | 1.1 | 79.2 | 100.0 |
| 50 | SucxFDT | 33.6 | 6.1 | 28.2 | 3.6 | 41.7 | 83.3 |
| 51 | SanxATf | 33.6 | 2.9 | 29.0 | 2.9 | 33.3 | 72.2 |
| 52 | SanxATf | 27.6 | 4.5 | 27.8 | 2.0 | 79.2 | 91.7 |
| 53 | SanxATf | 27.7 | 4.8 | 28.4 | 2.7 | 70.8 | 87.5 |
| 54 | SanxATf | 25.0 | 0.8 | 26.8 | 0.5 | 87.5 | 100.0 |
| 55 | FDTxSuc | 26.5 | 4.2 | 27.9 | 2.8 | 80.8 | 88.2 |
| 56 | FDTxSuc | 28.3 | 3.8 | 29.2 | 3.2 | 75.0 | 87.5 |
| 57 | SanxATf | 27.2 | 2.0 | 27.4 | 2.5 | 75.0 | 95.8 |
| 58 | SanxATf | 26.4 | 2.5 | 27.6 | 1.2 | 70.8 | 91.7 |
| 59 | SanxATf | 25.9 | 2.9 | 28.8 | 1.5 | 83.3 | 91.7 |
| 60 | FDTxSuc | 30.2 | 4.3 | 30.0 | 2.8 | 58.3 | 74.3 |
| 61 | FDTxSuc | 29.1 | 3.7 | 29.0 | 1.2 | 75.0 | 95.8 |
| 62 | SanxATf | 26.1 | 3.2 | 27.5 | 1.8 | 83.3 | 91.7 |
| 63 | SanxATf | 30.0 | 3.8 | 29.5 | 1.8 | 54.2 | 87.5 |
| 64 | FDTxSuc | 29.0 | 3.0 | 29.8 | 1.4 | 75.0 | 83.3 |
| 65 | SucxFDT | 26.1 | 3.1 | 27.4 | 1.3 | 87.5 | 95.8 |
| 66 | SanxATf | 27.7 | 3.0 | 27.6 | 0.7 | 87.5 | 100.0 |
| 67 | FDTxSuc | 25.2 | 0.1 | 29.7 | 0.2 | 73.7 | 85.7 |
| 68 | FDTxSuc | 26.6 | 3.9 | 29.0 | 2.9 | 70.8 | 83.3 |
| 69 | SanxATf | 27.8 | 3.5 | 27.2 | 1.2 | 79.2 | 95.8 |
| 70 | SanxATf | 25.8 | 5.4 | 26.8 | 1.2 | 79.2 | 100.0 |
| 71 | SanxATf | 28.3 | 3.1 | 29.5 | 2.0 | 83.3 | 83.3 |
| 72 | FDTxSuc | 30.5 | 5.4 | 29.9 | 4.4 | 45.0 | 61.6 |
| 73 | FDTxSuc | 27.5 | 1.2 | 28.9 | 1.0 | 83.3 | 91.7 |
| 74 | FDTxSuc | 28.6 | 5.6 | 27.9 | 0.7 | 62.5 | 100.0 |
| 75 | FDTxSuc | 28.9 | 6.3 | 27.7 | 2.6 | 62.5 | 91.3 |
| 76 | SucxFDT | 29.6 | 5.1 | 28.5 | 1.0 | 79.2 | 100.0 |
| 77 | FDTxSuc | 28.0 | 3.0 | 27.4 | 4.6 | 68.3 | 66.7 |
| 78 | SanxATf | 27.7 | 4.1 | 28.0 | 1.3 | 83.3 | 100.0 |
| 79 | SanxATf | 29.2 | 4.6 | 28.3 | 1.7 | 70.8 | 95.8 |
| 80 | SanxATf | 28.4 | 3.8 | 26.8 | 0.8 | 66.7 | 100.0 |
| 81 | SanxATf | 27.3 | 3.3 | 27.1 | 0.8 | 91.7 | 100.0 |
| 82 | SanxATf | 29.1 | 3.5 | 28.7 | 1.2 | 83.3 | 91.7 |
| 83 | FDTxSuc | 29.6 | 6.4 | 28.7 | 2.2 | 62.5 | 87.5 |
| 84 | SanxATf | 28.8 | 4.0 | 29.4 | 2.1 | 65.2 | 87.5 |
| 85 | SucxFDT | 25.7 | 1.4 | 27.5 | 2.4 | 87.5 | 91.7 |
| 86 | SanxATf | 26.5 | 2.5 | 27.0 | 0.9 | 83.3 | 100.0 |
| 87 | Hamlin sweet orange | 25.2 | 1.8 | 26.4 | 1.1 | 83.3 | 94.4 |
| 88 | Navel sweet orange | 24.7 | 1.4 | 26.2 | 1.2 | 89.3 | 100.0 |
| 89 | Argentina trifoliate | 36.4 | 4.1 | 37.8 | 2.8 | 25.0 | 15.0 |
| 90 | Flying Dragon trifoliate | 38.3 | 2.8 | 38.1 | 3.7 | 8.3 | 7.6 |
| 91 | Large Flower trifoliate | 37.6 | 3.1 | 36.5 | 3.9 | 15.6 | 27.9 |
| 92 | Pomeroy trifoliate | 36.7 | 3.3 | 35.6 | 4.9 | 22.9 | 33.5 |
| 93 | Rich 16-6 trifoliate | 36.5 | 4.7 | 37.2 | 4.6 | 8.3 | 12.9 |
| 94 | Rubidoux trifoliate | 36.1 | 5.0 | 36.7 | 4.3 | 25.0 | 25.0 |
| 95 | Volkamer lemon | 29.3 | 3.1 | 29.8 | 2.6 | 78.1 | 80.0 |
