# Supplementary material for: Construction of High-Density Genetic Maps and Detection of QTLs Associated With Huanglongbing Tolerance in Citrus
Source: Front Plant Sci. 2018 Nov 27;9:1694. doi: 10.3389/fpls.2018.01694 (PMC6278636; doi:10.3389/fpls.2018.01694)
Supplement: FILE S4 — Phenotypic data of two traits for the evaluation of HLB disease in 86 F1 progenies and nine control varieties. [file Table_4.docx]

**Supplementary File S4** Phenotypic data of two traits for the evaluation of HLB disease in 86 F_1_ progenies and nine control varieties. The phenotypic traits Foliar Symptom and Canopy Damage were evaluated basing on a 6-point scale visual rating. Each individual have eight replicate trees.

| Ind code | Cross type | Foliar-sym 2015 |  | Foliar-sym 2016 |  | Canopy-dam 2015 |  | Canopy-dam 2016 |  |
| --- | --- | --- | --- | --- | --- | --- | --- | --- | --- |
|  |  | Mean | SD | Mean | SD | Mean | SD | Mean | SD |
| 1 | SanxATf | 3.69 | 0.56 | 3.06 | 0.50 | 3.63 | 0.58 | 3.69 | 0.75 |
| 2 | SanxATf | 3.06 | 0.92 | 2.88 | 0.69 | 1.31 | 0.46 | 1.88 | 0.35 |
| 3 | SanxATf | 2.69 | 0.90 | 3.06 | 0.32 | 2.06 | 0.50 | 2.13 | 0.35 |
| 4 | SanxATf | 3.50 | 0.38 | 3.06 | 0.73 | 3.50 | 0.27 | 3.69 | 0.46 |
| 5 | SanxATf | 3.38 | 0.44 | 2.75 | 0.46 | 2.13 | 0.58 | 2.19 | 0.37 |
| 6 | FDTxSuc | 4.19 | 0.50 | 3.13 | 0.83 | 2.31 | 0.37 | 2.44 | 0.56 |
| 7 | SucxFDT | 3.50 | 0.71 | 2.88 | 0.69 | 1.75 | 0.38 | 2.00 | 0.00 |
| 8 | FDTxSuc | 3.67 | 0.58 | 3.83 | 0.68 | 4.08 | 0.58 | 3.92 | 0.49 |
| 9 | SucxFDT | 2.31 | 0.50 | 2.44 | 0.50 | 1.56 | 0.32 | 2.06 | 0.18 |
| 10 | SanxATf | 2.38 | 0.23 | 2.13 | 0.52 | 2.56 | 0.62 | 2.38 | 0.58 |
| 11 | SanxATf | 2.92 | 0.26 | 2.50 | 0.45 | 3.17 | 0.26 | 3.08 | 0.74 |
| 12 | SanxATf | 3.69 | 0.50 | 2.94 | 0.78 | 3.69 | 0.65 | 3.56 | 0.56 |
| 13 | SanxATf | 2.69 | 0.62 | 3.44 | 0.42 | 3.13 | 0.52 | 3.13 | 0.64 |
| 14 | SucxFDT | 3.39 | 0.75 | 3.21 | 0.64 | 2.64 | 0.63 | 2.93 | 0.35 |
| 15 | FDTxSuc | 3.61 | 0.56 | 3.36 | 0.38 | 2.79 | 0.76 | 3.14 | 0.85 |
| 16 | FDTxSuc | 4.39 | 0.24 | 4.43 | 0.45 | 3.07 | 0.84 | 3.50 | 0.50 |
| 17 | SanxATf | 3.44 | 0.37 | 2.88 | 0.44 | 2.56 | 0.50 | 2.75 | 0.27 |
| 18 | SanxATf | 4.19 | 0.50 | 3.00 | 0.60 | 2.56 | 0.82 | 2.56 | 0.73 |
| 19 | FDTxSuc | 4.38 | 0.35 | 2.88 | 0.64 | 1.81 | 0.46 | 1.94 | 0.32 |
| 20 | SucxFDT | 3.38 | 0.44 | 2.50 | 0.38 | 2.31 | 0.53 | 2.25 | 0.53 |
| 21 | FDTxSuc | 3.38 | 0.75 | 3.75 | 0.29 | 3.25 | 0.50 | 3.38 | 0.48 |
| 22 | SucxFDT | 2.38 | 0.74 | 2.94 | 0.32 | 2.25 | 0.38 | 2.50 | 0.46 |
| 23 | SanxATf | 3.06 | 0.46 | 3.00 | 0.46 | 2.19 | 0.59 | 2.00 | 0.71 |
| 24 | SanxATf | 2.69 | 0.32 | 3.06 | 0.42 | 2.50 | 0.38 | 2.44 | 0.42 |
| 25 | SucxFDT | 2.69 | 0.32 | 2.75 | 0.38 | 1.81 | 0.26 | 2.00 | 0.00 |
| 26 | SanxATf | 3.44 | 0.53 | 3.19 | 0.65 | 2.75 | 0.46 | 2.50 | 0.38 |
| 27 | SanxATf | 1.50 | 0.46 | 2.25 | 0.46 | 1.63 | 0.44 | 1.94 | 0.32 |
| 28 | FDTxSuc | 3.38 | 0.58 | 3.69 | 0.37 | 2.25 | 0.38 | 2.81 | 0.26 |
| 29 | FDTxSuc | 4.18 | 0.35 | 4.07 | 0.35 | 3.50 | 0.58 | 3.93 | 0.45 |
| 30 | SanxATf | 3.56 | 0.65 | 3.56 | 0.62 | 2.44 | 0.42 | 2.19 | 0.65 |
| 31 | SanxATf | 3.06 | 0.53 | 3.25 | 0.53 | 2.38 | 0.69 | 2.56 | 0.68 |
| 32 | SucxFDT | 3.33 | 0.66 | 3.00 | 0.55 | 3.67 | 0.41 | 3.42 | 0.38 |
| 33 | SucxFDT | 3.63 | 0.52 | 3.19 | 0.46 | 2.50 | 0.38 | 2.75 | 0.38 |
| 34 | SanxATf | 3.13 | 0.52 | 2.88 | 0.35 | 2.69 | 0.46 | 2.50 | 0.53 |
| 35 | FDTxSuc | 2.25 | 0.41 | 1.29 | 0.49 | 0.86 | 0.48 | 0.79 | 0.49 |
| 36 | FDTxSuc | 3.75 | 0.46 | 3.44 | 0.50 | 2.25 | 0.53 | 2.13 | 0.35 |
| 37 | FDTxSuc | 4.19 | 0.62 | 3.69 | 0.53 | 2.00 | 0.38 | 2.44 | 0.42 |
| 38 | SucxFDT | 3.13 | 0.64 | 2.75 | 0.65 | 2.31 | 0.46 | 2.31 | 0.37 |
| 39 | SanxATf | 3.81 | 0.42 | 2.94 | 0.50 | 2.38 | 0.44 | 2.38 | 0.44 |
| 40 | SanxATf | 1.31 | 0.42 | 2.81 | 0.53 | 1.94 | 0.50 | 2.44 | 0.32 |
| 41 | SanxATf | 2.00 | 0.60 | 2.69 | 0.80 | 2.38 | 0.69 | 2.56 | 0.68 |
| 42 | SanxATf | 2.63 | 0.74 | 2.94 | 0.42 | 2.00 | 0.53 | 2.38 | 0.35 |
| 43 | FDTxSuc | 3.81 | 0.42 | 3.25 | 0.46 | 2.69 | 0.53 | 2.56 | 0.82 |
| 44 | SanxATf | 3.50 | 0.60 | 2.69 | 0.37 | 2.19 | 0.26 | 1.94 | 0.18 |
| 45 | SucxFDT | 2.63 | 0.23 | 2.44 | 0.56 | 1.63 | 0.52 | 1.69 | 0.46 |
| 46 | SanxATf | 4.00 | 0.46 | 3.44 | 0.50 | 3.19 | 0.88 | 3.19 | 0.70 |
| 47 | SanxATf | 2.81 | 0.32 | 3.06 | 0.42 | 3.56 | 0.82 | 3.44 | 0.56 |
| 48 | SanxATf | 3.32 | 0.45 | 2.71 | 0.39 | 3.21 | 0.57 | 3.21 | 0.49 |
| 49 | SanxATf | 3.69 | 0.56 | 3.00 | 0.60 | 2.38 | 0.35 | 2.56 | 0.42 |
| 50 | SucxFDT | 3.82 | 0.53 | 4.07 | 0.19 | 3.43 | 0.61 | 4.14 | 0.38 |
| 51 | SanxATf | 3.25 | 0.55 | 2.83 | 0.61 | 3.17 | 0.75 | 2.92 | 0.92 |
| 52 | SanxATf | 2.38 | 0.23 | 3.50 | 0.65 | 3.00 | 0.60 | 3.19 | 0.26 |
| 53 | SanxATf | 3.50 | 0.46 | 3.31 | 0.53 | 3.31 | 0.46 | 3.56 | 0.50 |
| 54 | SanxATf | 3.06 | 0.59 | 3.19 | 0.37 | 2.44 | 0.50 | 2.75 | 0.38 |
| 55 | FDTxSuc | 3.55 | 0.85 | 3.58 | 0.58 | 3.68 | 0.35 | 2.96 | 0.56 |
| 56 | FDTxSuc | 4.31 | 0.32 | 4.06 | 0.56 | 2.88 | 0.79 | 3.00 | 0.71 |
| 57 | SanxATf | 1.75 | 0.71 | 2.25 | 0.53 | 1.94 | 0.62 | 1.94 | 0.73 |
| 58 | SanxATf | 3.00 | 0.38 | 3.13 | 0.35 | 3.31 | 0.53 | 3.19 | 0.59 |
| 59 | SanxATf | 3.38 | 0.35 | 3.63 | 0.69 | 4.13 | 0.44 | 4.00 | 0.46 |
| 60 | FDTxSuc | 2.46 | 0.26 | 3.04 | 0.32 | 1.56 | 0.32 | 1.41 | 0.46 |
| 61 | FDTxSuc | 1.88 | 0.44 | 1.88 | 0.44 | 1.06 | 0.32 | 1.13 | 0.35 |
| 62 | SanxATf | 2.44 | 0.84 | 2.56 | 0.68 | 2.50 | 0.76 | 2.63 | 0.79 |
| 63 | SanxATf | 3.25 | 0.71 | 2.94 | 0.32 | 2.44 | 0.68 | 2.38 | 0.52 |
| 64 | FDTxSuc | 4.19 | 0.50 | 3.81 | 0.65 | 2.63 | 0.35 | 2.31 | 0.46 |
| 65 | SucxFDT | 3.44 | 0.84 | 3.13 | 0.35 | 1.31 | 0.26 | 1.75 | 0.27 |
| 66 | SanxATf | 2.63 | 0.74 | 2.75 | 0.46 | 2.50 | 0.71 | 2.31 | 0.46 |
| 67 | FDTxSuc | 3.75 | 0.71 | 4.50 | 0.71 | 2.25 | 1.06 | 4.00 | 1.41 |
| 68 | FDTxSuc | 2.94 | 0.84 | 2.25 | 0.38 | 1.88 | 0.23 | 2.00 | 0.27 |
| 69 | SanxATf | 3.39 | 0.56 | 2.50 | 0.71 | 3.50 | 1.22 | 2.86 | 0.90 |
| 70 | SanxATf | 2.63 | 0.52 | 3.06 | 0.32 | 2.31 | 0.53 | 2.38 | 0.52 |
| 71 | SanxATf | 3.56 | 0.59 | 2.81 | 0.46 | 2.44 | 0.32 | 2.19 | 0.46 |
| 72 | FDTxSuc | 2.43 | 0.35 | 1.94 | 0.32 | 1.63 | 0.44 | 1.81 | 0.46 |
| 73 | FDTxSuc | 4.18 | 0.67 | 3.79 | 0.39 | 2.64 | 0.75 | 2.36 | 0.56 |
| 74 | FDTxSuc | 4.32 | 0.35 | 4.21 | 0.39 | 2.86 | 0.69 | 2.93 | 1.02 |
| 75 | FDTxSuc | 3.92 | 0.61 | 3.92 | 0.20 | 3.00 | 0.55 | 3.17 | 0.26 |
| 76 | SucxFDT | 3.69 | 0.68 | 3.69 | 0.53 | 1.75 | 0.65 | 2.50 | 0.46 |
| 77 | FDTxSuc | 3.54 | 0.53 | 3.64 | 0.42 | 3.51 | 0.53 | 2.95 | 0.38 |
| 78 | SanxATf | 2.75 | 0.89 | 3.19 | 0.46 | 3.69 | 0.75 | 3.75 | 0.65 |
| 79 | SanxATf | 2.31 | 0.50 | 2.19 | 0.46 | 1.94 | 0.42 | 1.81 | 0.37 |
| 80 | SanxATf | 3.44 | 0.53 | 2.38 | 0.44 | 2.13 | 0.44 | 1.88 | 0.44 |
| 81 | SanxATf | 1.56 | 0.26 | 1.75 | 0.27 | 1.25 | 0.38 | 1.50 | 0.38 |
| 82 | SanxATf | 2.63 | 0.64 | 2.63 | 0.44 | 3.31 | 0.37 | 2.63 | 0.58 |
| 83 | FDTxSuc | 4.69 | 0.18 | 3.44 | 0.56 | 1.63 | 0.35 | 1.94 | 0.18 |
| 84 | SanxATf | 2.83 | 0.74 | 3.33 | 0.41 | 3.42 | 0.80 | 3.08 | 0.74 |
| 85 | SucxFDT | 3.38 | 0.44 | 2.69 | 0.70 | 3.88 | 0.69 | 3.69 | 0.70 |
| 86 | SanxATf | 2.75 | 0.38 | 3.13 | 0.58 | 2.56 | 0.68 | 2.50 | 0.71 |
| 87 | Hamlin sweet orange | 4.08 | 0.26 | 3.75 | 0.89 | 3.83 | 0.75 | 3.67 | 0.82 |
| 88 | Navel sweet orange | 3.92 | 0.29 | 3.63 | 0.48 | 4.50 | 0.87 | 4.38 | 0.75 |
| 89 | Argentina trifoliate | 1.13 | 0.35 | 1.38 | 0.44 | 1.31 | 0.46 | 1.06 | 0.18 |
| 90 | Flying Dragon trifoliate | 1.68 | 0.98 | 1.93 | 0.35 | 1.97 | 0.84 | 2.04 | 0.94 |
| 91 | Large Flower trifoliate | 1.56 | 0.70 | 1.44 | 0.18 | 1.25 | 0.38 | 1.25 | 0.38 |
| 92 | Pomeroy trifoliate | 1.19 | 0.32 | 1.38 | 0.35 | 1.31 | 0.46 | 1.00 | 0.00 |
| 93 | Rich 16-6 trifoliate | 1.44 | 0.37 | 1.19 | 0.26 | 1.44 | 0.42 | 1.06 | 0.18 |
| 94 | Rubidoux trifoliate | 1.25 | 0.27 | 1.38 | 0.35 | 1.50 | 0.46 | 1.13 | 0.23 |
| 95 | Volkamer lemon | 2.85 | 0.21 | 2.27 | 0.42 | 1.17 | 0.36 | 1.07 | 0.18 |
